# A Conserved TCRβ Signature Dominates a Highly Polyclonal T-Cell Expansion During the Acute Phase of a Murine Malaria Infection

**DOI:** 10.3389/fimmu.2020.587756

**Published:** 2020-11-23

**Authors:** Natasha L. Smith, Wiebke Nahrendorf, Catherine Sutherland, Jason P. Mooney, Joanne Thompson, Philip J. Spence, Graeme J. M. Cowan

**Affiliations:** Institute of Immunology and Infection Research, School of Biological Sciences, University of Edinburgh, Edinburgh, United Kingdom

**Keywords:** T-cell, TCR, immune-repertoire, single-cell, *Plasmodium chabaudi*, malaria

## Abstract

CD4^+^ αβ T-cells are key mediators of the immune response to a first *Plasmodium* infection, undergoing extensive activation and splenic expansion during the acute phase of an infection. However, the clonality and clonal composition of this expansion has not previously been described. Using a comparative infection model, we sequenced the splenic CD4^+^ T-cell receptor repertoires generated over the time-course of a *Plasmodium chabaudi* infection. We show through repeat replicate experiments, single-cell RNA-seq, and analyses of independent RNA-seq data, that following a first infection - within a highly polyclonal expansion - T-effector repertoires are consistently dominated by TRBV3 gene usage. Clustering by sequence similarity, we find the same dominant clonal signature is expanded across replicates in the acute phase of an infection, revealing a conserved pathogen-specific T-cell response that is consistently a hallmark of a first infection, but not expanded upon re-challenge. Determining the host or parasite factors driving this conserved response may uncover novel immune targets for malaria therapeutic purposes.

## Introduction

Although protective natural immunity against clinical malaria is slow to develop and requires years of repeated exposure (1), protection against severe disease is obtained after a more limited number of symptomatic infections (2, 3). The acquisition of this naturally acquired immunity is mediated by both antibody [reviewed in (4, 5)] and T-cell responses (6); the latter being crucial for B-cell class switching and affinity maturation. As well as guiding the humoral response, CD4^+^ T-cells play a key role in restricting the growth and pathogenesis of blood-stage *Plasmodium* through cytokine secretion and macrophage activation [reviewed in (7)]. However, the antigenic drivers and developmental dynamics underlying this naturally acquired immunity remain poorly understood, presenting major challenges for effective vaccine design.

In animal models of malaria, a *Plasmodium* infection in previously unexposed individuals initially produces a massive expansion of CD4^+^ T-cells in the spleen (8, 9), a major site of the developing immune response (10). The size of this response, together with the generation of a highly diverse range of cellular responses, suggests that the splenic expansion of CD4^+^ populations is highly polyclonal, as opposed to the expansion of a minor (oligoclonal) subset of the repertoire. However, it is not known whether this expansion is primarily a non-specific response, such as a result of cytokine-driven bystander activation, or whether it is dominated by antigen-specific responses generated through classic TCR-engagement mediated clonal expansion. Spectratyping (CDR3 length analysis) of T-cell receptor (TCR) β chain repertoires induced by the rodent malaria *Plasmodium berghei* has previously detected a unique TCRβ CDR3 length signature enhanced over the course of infection, suggesting that there may be a clonal response to specific antigenic peptides (11). In agreement with this, an expanded fraction of CD4^+^ T-cells and fast-responding cytokine secretors that respond to a secondary challenge has been observed following a *Plasmodium chabaudi (AJ)* infection in mice, indicating initial priming by the parasite, and the presence of pathogen-specific T-cells within the CD4^+^ T-cell population (9). Alternatively, there is evidence from *P. falciparum*, that the PfEMP1 binding domain, CIDR-1α, stimulates CD4^+^ T-cells non-specifically through TCR-independent pathways (12), and that regulatory T-cell (Treg) proliferation during an infection can be induced in an antigen non-specific manner (13). Non-specific proliferation of T-cells due to cross-reactivity in response to *P. falciparum* antigens has also been reported (14).

Overall, proliferation is likely to be a combination of activation dynamics. However, whether a detectable clonal malaria-specific CD4^+^ T cell response that is conserved between individuals, and thus a potential focal target for therapeutics, is induced, has not previously been demonstrated.

Advances in high-throughput TCR repertoire sequencing techniques now allow deep profiling of immune responses. This approach has been used to ascertain clonality of T-cell responses, identify expanded T-cell clones and determine if conserved or ‘public’ responses between individuals are generated following antigenic stimulation [reviewed in (15)]. Repertoire sequencing thus provides a novel, immune-focused approach to delineate the clonality of the developing immune response to a malaria infection.

Here, using bulk TCRβ repertoire sequencing we examine the dynamics and clonal structure of the splenic CD4^+^ T-cell repertoires generated during infection with the well-established mouse malaria model *Plasmodium chabaudi (AS)*. By comparing serially blood passaged (SBP) and recently mosquito-transmitted (MT) *P. chabaudi* infections, Spence et al. (8) demonstrated that vector transmission of *P. chabaudi* intrinsically modified parasite gene expression in asexual blood-stage parasites, eliciting an altered host immune response that in turn regulates parasite virulence. In this model, infection with SBP parasites leads to hyperparasitaemia with more severe disease during the acute phase of infection. In contrast, mosquito transmission (MT) attenuates parasite growth and virulence, through a mechanism associated with epigenetic reprogramming of the expression of the subtelomeric multigene families, including the variant surface antigen (VSA) family. We have used this comparative model to compare TCR repertoires integral to an immune response that rapidly controls parasite growth, against a less effective response that fails to control parasite replication and induces immunopathology (8, 16). We have sequenced the T-naive (T_N_), T-effector (T_E_), T-effector memory (T_EM_) and T-central memory (T_CM_) CD4^+^ splenic TCRβ repertoires elicited in mice over the time-course of both MT and SBP *P. chabaudi* infections. We report, for both infection types, that the T_E_ expansion seen during the acute phase of a *P. chabaudi* infection is highly polyclonal. However, within this diverse expansion, a conserved pathogen-specific response characterized by TRBV3 gene usage consistently dominates the effector repertoire following a first infection, and we further profile this response using single-cell RNA-seq.

## Materials and Methods

### Mice Infections

All procedures were carried out in accordance with UK Home Office regulations (Animals Scientific Procedures Act, 1986; project license number 70/8,546 and P04ABDCAA) and were approved by the Ethical Review Body of the University of Edinburgh. C57Bl/6 mice were bred and housed under specific pathogen free conditions at the University of Edinburgh and subjected to regular pathogen monitoring by sentinel screening. Mice were housed with at least one companion in individually ventilated cages furnished with autoclaved woodchip, fun tunnel and tissue paper at 21°C ± 2°C under a reverse light-dark cycle (light, 19.00 – 07.00; dark, 07.00 – 19.00) at a relative humidity of 55% ± 10%. *P. chabaudi (AS)* parasites were obtained from the European Malaria Reagent Repository at the University of Edinburgh. Eight- to 10-week-old C57Bl/6 female mice were infected with *P. chabaudi (AS)* by intra-peritoneal injection of 1x10^5^ parasitized erythrocytes that had either been maintained by serial blood-passage over a high number of generations (SBP) or undergone a single passage following mosquito transmission (MT) as per Spence et al. (17). Each transmission group consisted of five cages of five mice, with five unchallenged mice from the same cohort used as experimental controls. Mice (n=5 per transmission group) were euthanized on days 6, 10, 20, 40, and 60 post-infection. In a repeat experiment, mice (n=4 per time-point) were infected with MT *P. chabaudi (AS)-GFP* (18) by intra-peritoneal injection of 1x10^5^ parasitized erythrocytes, and were euthanized at days 4, 7, 11, and 14 post-infection. For the re-challenge experiment, mice were infected with MT *P. chabaudi (AS)-GFP* by intra-peritoneal injection of 1x10^5^ parasitized erythrocytes. Mice (n=4) underwent a homologous re-challenge at day 60 post infection and were euthanized 7 days post re-challenge (day 67). To eliminate chronic infection before re-challenge, 0.288 mg/ml of chloroquine diphosphate salt (Sigma), supplemented with glucose for palatability, was dissolved in drinking water daily for 10 days, from day 30 to day 40 post-infection (19). For the single-cell RNA-seq experiment, mice (n=2) were infected with MT *P. chabaudi (AS)-GFP* by intra-peritoneal injection of 1x10^5^ parasitized erythrocytes, and euthanized at day 7 post-infection.

### Cell Sorting

CD4^+^ splenic T-cell populations of interest were isolated by FACS using a BD FACSAria III instrument, according to gates described by Spence et al. (8): T_N_ (CD62L^+^ CD127^+^), T_E_ (CD62L^-^ CD127^-^), T_EM,_ (CD44HI CD127^+^ CD62L^-^) (20), and T_CM_ (CD44HI CD127^+^ CD62L^+^) (**Supplementary Figure 1**). For the repeat and re-challenge experiments, only T_E_ and T_N_ populations were isolated. Cells were sorted into 50 µl FACS buffer and stored at -80°C until processing. For the single-cell experiment, CD3^+^ CD4^+^ splenocytes were sorted in to 100 µl 0.04% BSA in PBS, to generate the single-cell suspension required for 10x Genomics sequencing.

### Unbiased Bulk TCR Amplification and Sequencing

RNA was extracted from CD4^+^ splenic T-cell populations of interest using Dynabeads mRNA purification kit (ThermoFisher Scientific). cDNA was synthesized from each RNA preparation by adding the following to each sample: 4 µl First-strand Buffer, 2 µl 10mM dNTP mix, 2 µl 20mM DTT and 2 µl SMART-PTO2 oligo (5’ AAGCAGTGGTATCAACGGAGAGTACATrGrGr 3’), 0.5 µl RNase inhibitor (Clontech 2313A), and 2 µl (100 U/µl) SMARTScribe reverse transcriptase (Clontech). For the repeat experiments, unique molecular identifiers (UMIs) (21) were incorporated during cDNA synthesis by replacing the template-switch oligo with 2 µl SMARTNNN oligo (5’AAGCAGUGGTAUCAACGCAGAGUNNNNUNNNNUNNNNUCTTrGrGrG 3’). Samples were then incubated at 42°C for 70 min, before the reaction was terminated by heating at 70°C for 10 min. cDNA synthesized with SMARTNNN oligos were treated with 1 μl of Uracil DNA glycosylase (5 U/μl, New England Biolabs) and incubated for 15 min at 37°C. PCR was then used to generate TCRβ V-region amplicons, using indexed forward primers composed of the SMART synthesis oligo sequence fused to a P7 Illumina tag, and a reverse primer within the TCR-C region fused to a P5 Illumina tag (P5-mTCRBrev3: 5’ AATGATACGGCGACCACCGAGATCTACACCTTGGGTGGAGTCACATTTCT 3’). Amplified products were purified by extraction from excised agarose gel bands. Single-end 1x400bp and asymmetric 100bp+400bp (to incorporate UMIs) sequencing was performed on an Illumina MiSeq platform.

### Bulk TCR Repertoire Analyses

Bulk TCR sequence data was initially processed using MiGEC (22) and MiXCR (23) software with default settings. Samples were excluded from further analyses if the repertoire contained fewer than 10,000 total reads after processing, as this was indicative of poor sample preservation or preparation. A combination of custom pipelines of Python scripts and VDJtools software (24) was used to analyze and plot the MiXCR output. Statistical analyses were performed using SciPy Python software (25). A TCR clone was defined by 100% amino acid sequence identity of the CDR3 region, and IMGT nomenclature used for gene usage. Only in-frame (functional) CDR3s were analyzed. A modified version of the Swarm algorithm (26) was used to cluster highly homologous CDR3 amino sequences, with identical V-gene usage, within 1 amino-acid mismatch of each other. The Gliph2 package (27) was also used to identify enriched amino acid motifs within the contact region of CDR3 sequences; the unchallenged T-naïve repertoires were used to make custom murine reference files for this. Network analyses was undertaken using Gephi (28) software (v0.9.2). Generation probability of TCRs (Pgen) was calculated using OLGA (29).

### Publicly Available RNA-Seq Data Analyses

Raw FASTQ files from RNA-seq data obtained from the spleens of C57Bl/6 mice infected with blood-stage *Plasmodium chabaudi (AS)* and *Plasmodium chabaudi (CB)* were downloaded from the ArrayExpress archive: ENA - ERP004042 and ENA - ERP005730 respectively. For comparison of infection with other pathogens, raw FASTQ files of RNA-seq data obtained from whole blood of C57Bl/6 mice infected with a variety of pathogens, were downloaded from the NCBI short read archive, under accession SRR7821557. Normalized Trbv3 gene expression values were also obtained from Singhania et al. (30). RNA-seq data was aligned using MiXCR (23) software, and a combination of custom pipelines of Python scripts and VDJtools software (24) was used to analyze and plot the MiXCR output.

### Single-Cell Sequencing, Data Processing, and Analyses

Two barcoded cDNA libraries were prepared from sorted samples using the Chromium Single Cell 5’ Library Kit v2 (31). Full length V(D)J segments were enriched from amplified cDNA with primers specific to the TCR constant region using the Chromium Single Cell V(D)J Enrichment Kit – Mouse T-Cell. Sequencing was performed using the High-Output v2.5 Kit on a NextSeq 550 platform. Initial processing of sequence files, including mapping of reads to the mouse reference genome (GRCm38), generation of count matrixes and assembly of TCR alpha and beta chains, was carried out using CellRanger 3.1.0. To exclude potential multiplets, poor quality cells or non T-cells, single T-cells were identified by the expression of a single productive beta chain. Barcodes lacking a beta chain or assigned to multiple were excluded, leaving data from 3,333 single T-cells (1,658 and 1,675 from mouse 1 and 2 respectively). Downstream analyses were performed in R using Seurat 3.1.5 (32). Genes expressed in fewer than three cells, as well as all *Trav/j* and *Trbv/d/j* genes were excluded. Cells expressing fewer than 200, or over 3,000 genes and/or more than 5% mitochondrial genes were removed. The filtered matrix was normalized using Seurat’s LogNormalize with default parameters and the top 2,000 variable genes were identified using the FindVariableFeatures ‘vst’ method, before centering and scaling of the matrix. Dimensionality reduction by PCA was carried out and the top 30 principal components were used as input for graph-based clustering. Clusters were visualized by UMAP. A small, poorly *Cd4*-expressing cluster was identified, and these cells were excluded as contaminants. The above normalization and clustering steps were repeated with the remaining 2,976 cells (1,491 and 1,485 from mouse 1 and 2 respectively). Differential gene expression analysis using the Wilcoxon rank sum test through FindAllMarkers was used to identify marker genes for each cluster.

## Results

Mice were infected with *P. chabaudi (AS)* parasitized erythrocytes from donor mice infected with either recently MT or SBP parasites, and followed for 60 days of infection. For repeat experiments, only MT parasites were used, as these represent a less artificial experimental model. At each time point, spleens were harvested and CD4^+^ splenic T-cells populations of interest were isolated by FACS before RNA was extracted, reverse transcribed, and TCRβ chains amplified and sequenced.

### TRBV3 Gene Usage Dominates a Highly Polyclonal T-Effector Expansion

**Figure 1:**
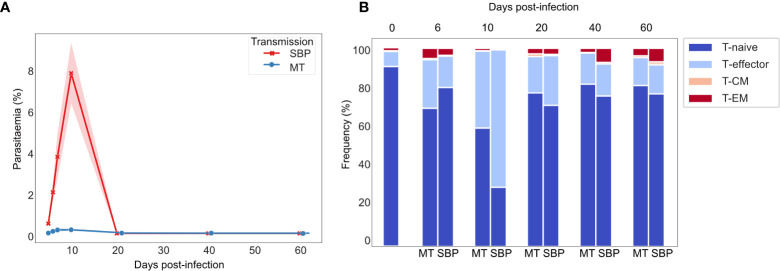
Dynamics of *P. chabaudi* infection: T-effector expansion coincides with peak parasitemia. **(A)** Parasitaemia of C57Bl/6 mice infected with either serially blood passaged (SBP) (red) or recently MT (blue) 5 x10^5^ *P. chabaudi* parasitized erythrocytes, n=5 mice per infection type per time point, shaded area depicts 95% CI. **(B)** Phenotypic profiling of CD4^+^ T cells as determined by FACS. Representative frequencies over the time course of infection of T-naïve CD4^+^ T cells (CD62L^+^ CD127^+^), T-effector CD4^+^ T cells (T_E_) (CD62L^-^ CD127^-^), effector memory (T_EM_) (CD44HI CD127^+^ CD62L^-^) and central memory (T_CM_) (CD44HI CD127^+^ CD62L^+^) CD4^+^T cells. Unchallenged control mice are also represented.

**Figure 2:**
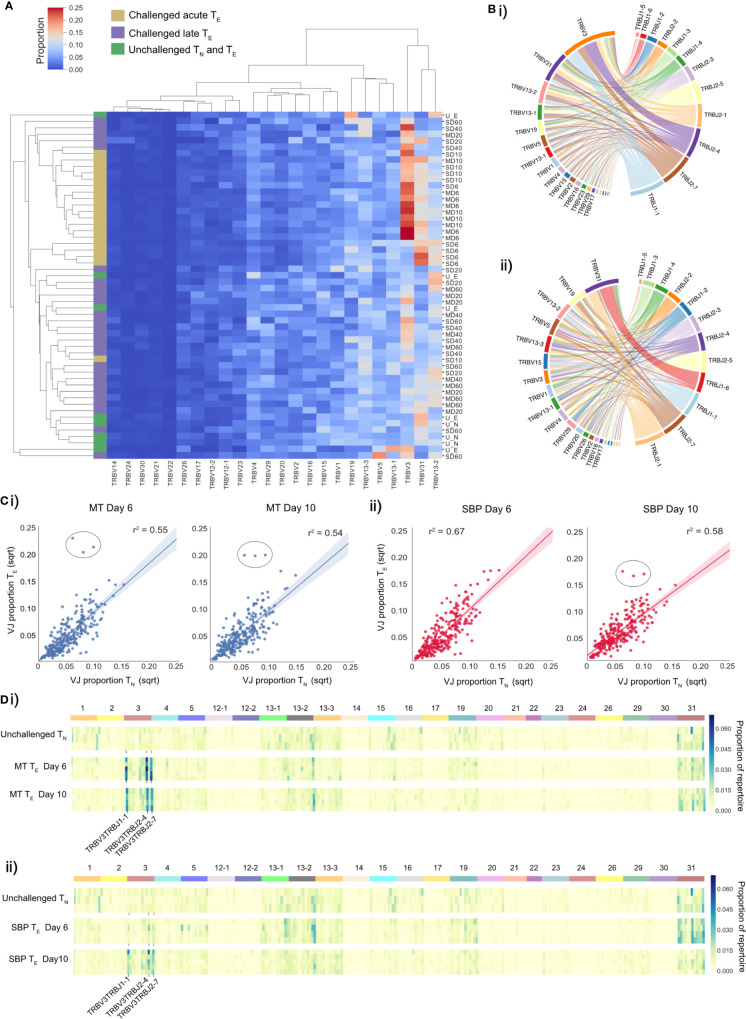
T_E_ repertoires have dominant TRBV3 gene usage during the T_E_ expansion in acute phase of a *P. chabaudi* infection. **(A)** Clustermap displays TRBV gene proportional usage for individual splenic CD4^+^ T-cell receptor (TCR) repertoires from challenged acute T_E_ repertoires (gold), challenged late phase T_E_ repertoires (purple) and unchallenged T_N_ and T_E_ repertoires (green). Each column is a unique TRBV gene and each row an individual repertoire. **(B)** Circos plots show V/J gene usage from a representative repertoire of i) a challenged MT T_E_ repertoire at day 6 post-infection and ii) an unchallenged T_N_ repertoire. Band width is proportional to usage frequency. **(C)** Mean proportion of each V/J allelic combination in unchallenged T_N_ repertoires versus challenged T_E_ repertoires at days 6 and 10 post-infection for mice infected with (i) MT parasites (blue) and (ii) SBP parasites (red). Each point represents a unique V/J combination. **(D)** Heatmaps depict proportional usage of each V/J allelic combination (columns) for individual replicate mice (rows) for (i) unchallenged T_N_ repertoires and acute T_E_ MT repertoires and (ii) unchallenged T_N_ repertoires and acute T_E_ SBP repertoires. Data for both days 6 and 10 post-infection are displayed. Horizontal color bar indicates TRBV gene used in the V/J combination.

Consistent with previously published data for *P. chabaudi* (8, 33), CD4^+^ splenic T-effectors (T_E_) reached maximum levels of expansion in the acute phase of infection, increasing by up to 10-fold. Expansion coincided with the peak of parasitemia and contracted back to pre-challenged levels between days 20 and 40 post-infection (**Figures 1A, B**). We first hypothesized that if the T_E_ expansion in the acute phase of infection was solely the result of non-specific activation, V gene usage and V/J allele usage within the T_E_ repertoire would mirror that of T_N_ repertoires, despite the vast cellular proliferation. Thus, there would be no change in the distribution of V or V/J allele usage post parasite-challenge. However, a distinct increase in TRBV3 gene usage was observed during the acute phase of infection, differentiating challenged T_E_ repertoires from both unchallenged T_N_ and T_E_ repertoires, and from challenged T_E_ repertoires at later time-points (**Figures 2A, B**, **Supplementary Figure 2**). For MT infections during the acute phase, TRBV3 encoded on average 23.7% (± 2.03 95% CI) of the effector repertoire at day 6 and 21.6% (± 2.21 95% CI) at day 10 post-infection, compared to only 7.6% (± 0.47, 95% CI) of the unchallenged naïve repertoire. This finding was repeated in a second independent experiment (**Supplementary Figure 3**), and through analysis of publicly available RNA-seq data for *P. chabaudi (AS)* and *P. chabaudi (CB)* (accession E-ERAD-221 and E-ERAD-289 respectively, **Supplementary Figure 4**). This increase in TRBV3 usage was more delayed in SBP challenged repertoires, and not apparent until day 10 post-challenge, where at its peak it encoded 17.2% (± 2.54, 95% CI) of the T_E_ repertoire.

For V/J allele combination usage, an overall strong positive correlation between challenged T_E_ and unchallenged T_N_ repertoires was evident during the effector expansion, indicating a highly polyclonal response with broad expansion of the naïve precursor pool. However, three specific TRBV3/J allelic combinations, TRBV3-TRBJ1-1, TRBV3-TRBJ2-4 and TRBV3-TRBJ2-7, were disproportionately increased in challenged T_E_ repertoires of mice infected with MT parasites at both days 6 and day 10, and for SBP infections by day 10 post-infection (**Figure 2C**). This specific V/J usage was conserved across all individual replicate mice infected with MT parasites during the acute phase of infection (**Figure 2D**) and was evident in the repeat experiment (**Supplementary Figures 5A, Biii**). During the late phase of infection, as the T_E_ population contracted, this conserved V/J signature was lost in both infection types (**Supplementary Figures 5Bi, Bii**).

**Figure 3:**
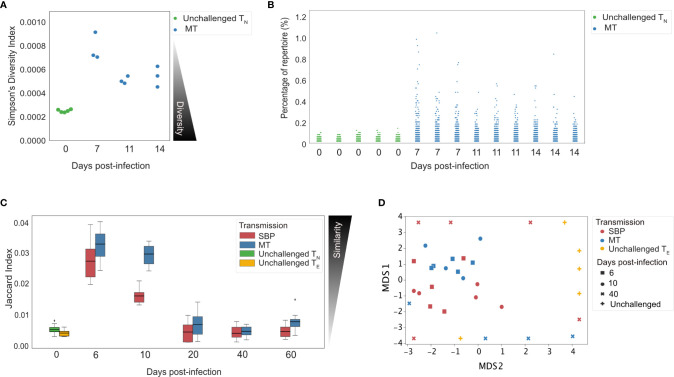
Splenic T_E_ repertoires elicited by *Plasmodium chabaudi* are highly diverse. **(A)** Simpson’s Diversity Index (SI) for UMI-size matched unchallenged T_N_ (green) and acute MT T_E_ repertoires (blue). SI varies from 0 to 1, and for T-cell receptor (TCR) repertoires represents the probability that identical TCRs (as determined by identical CDR3 amino acid sequence) will be drawn from the repertoire with two independent draws. SI of 0 therefore represents maximal diversity. **(B)** Each point in the strip plot represents a clone and the percentage of repertoire they occupy, for individual replicate mice for T_N_ unchallenged repertoires (green) and MT T_E_ repertoires (blue). Data for **(A, B)** was normalized by down-sampling to 5000 UMI. **(C)** Similarity between replicate repertoires is demonstrated using the Jaccard index, a normalized sharing metric which represents the degree of clonal overlap between repertoires. Jaccard index varies form 0 (no similarity) to 1 (identical repertoires). T_E_ replicate repertoires are more similar to each other in the acute phase of infection for both infection types. **(D)** Multi-dimensional-scaling (MDS) analysis using Jaccard similarity index of T_E_ repertoires for unchallenged (yellow), MT (blue) and SBP (red) repertoires. Data for **(C, D)** was normalized by down-sampling to 10^4^ reads and calculated on weighted data to include clonotype frequency.

Changes in diversity of a TCR repertoire following pathogen exposure are indicative of the extent to which clonal expansion within a repertoire has occurred (34). To assess the diversity of the acute CD4^+^ T_E_ expansion response at the clonal level, repertoire diversity was calculated using Simpson’s diversity index on age-matched (35) and size-matched repertoires from the repeat experiment. This allowed us to sample an equal number of UMI-labeled cDNA molecules for the precise normalization required for comparing diversity metrics (36). Although unchallenged T_N_ repertoires were, as expected, significantly more diverse than challenged T_E_ repertoires (**Figure 3A**, p<0.01), the T_E_ repertoires after challenge were still highly polyclonal – with the most abundant clone taking up on average only 0.72% (± 0.11%) of the repertoire compared to 0.112% (± 0.01%) in T_N_ unchallenged repertoires (**Figure 3B**).

### T-Effector Repertoires Have Greater Similarity During Acute Infection

If a pathogen-specific response is elicited at the clonal level, we would expect challenged T_E_ repertoires of replicate mice to contain an increased number of shared clones, and to therefore be more similar to each other than unchallenged repertoires. To examine the degree of repertoire sharing between replicate mice, the Jaccard index, a similarity or ‘overlap’ metric was used, matching at the CDR3 amino acid sequence level. Over the course of infection, for each infection type, similarity between replicates was significantly altered (one-way ANOVA, MT: p<0.001, SBP: p<0.001) (**Figure 3C**), with replicates being more similar to each other in the acute phase of infection than at later time-points. MT repertoires were also more similar to each other during the acute phase than SBP infections (day 6: t=2.6, p=0.016, day 10: t=7.2, p<0.001). For both infection types, during the acute phase, replicate repertoires were more similar to each other than to unchallenged T_N_ (day 6: MT: t=15.13, p <0.001, SBP: t=11.7, p<0.001, day 10: MT: t=13.4, p<0.001, SBP: t=9.2, p<0.001) and unchallenged T_E_ repertoires (day 6: MT: t=15.1, p<0.001, SBP: t=11.7, p <0.001, day 10: MT: t=13.4, p<0.001, SBP: t=9.2, p<0.001). Randomly sampling the same number of sequences (10^4^) to produce size-matched repertoires did not alter this pattern of results, nor did size-matching the repeat UMI data (**Supplementary Figure 6**). Further exploration using multi-dimensional scaling (MDS) of size-matched repertoires, also indicated clustering of acute T_E_ repertoires for both infection types according to Jaccard similarity, with MT repertoires at day 6 and 10 most tightly co-clustered (**Figure 3D**).

**Figure 4:**
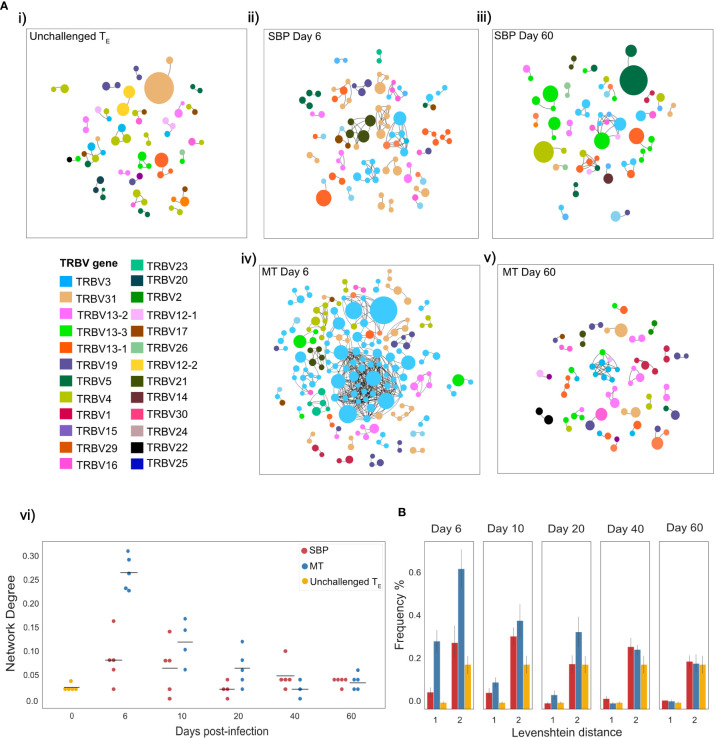
Mosquito-transmitted (MT) acute repertoires have greater clonal network connectivity. **(A)** (i–v) Networks showing the top 100 most abundant CDR3 amino acid sequences in replicate repertoires within a Levenshtein distance of 1 of each other. For T-cell receptors (TCRs) a Levenshtein distance of 1 represents 1 amino acid mismatch (insertion, deletion or substitution) between CDR3 sequences. Each node represents a TCR clone as defined by CDR3 amino acid sequence, with node size indicating proportion of repertoire occupied by clone. Nodes are colored according to TRBV-gene usage. An edge is drawn between nodes if within a Levenshtein distance of 1, with unconnected nodes not depicted, (vi) network degree (mean number of edges per node) for each individual T_E_ repertoire network. **(B)** Frequency (%) of individual CDR3 sequence pairwise-comparisons, within the top 100 most abundant CDR3 sequences of each individual T_E_ repertoire, that are within a Levenshtein distance of 1 and 2.

The TCR repertoire is a dynamic network, so examining similarity solely at the clonal level can fail to take in to account the degree of extended clonal networks that may be present. Despite not undergoing somatic hypermutation, T-cell repertoires have been shown to contain networks generated by sequence similarity, with CDR3 sequence similarity and thus network connectivity increased in antigen-experienced repertoires (37, 38). To explore connectivity between CDR3s in the T_E_ repertoires, network analysis was undertaken between the top 100 most abundant clones, using Levenshtein distance. Networks were constructed between replicate repertoires, by creating an edge between unique CDR3 sequences (nodes) if they were within a Levenshtein distance of one of each other (**Figure 4A**). Node degree (the average number of edges per node within network) for each individual indicated a higher degree of connectivity for both infection types in the acute stage of infection compared to unchallenged T_E_ repertoires (Mann-Whitney U, (day 6: MT: p<0.01, SBP: p=0.022, day10: MT: p<0.01, SBP: p=0.045) (**Figure 4Avi**)). MT repertoires also tended to have a higher node degree than SBP repertoires at days 6, 10 and 20 post-infection, although significance was not detected at day 10 (Mann-Whitney U, day 6: p<0.01, day 10: p=0.088, day 20: p=0.039). Within each individual, there was also an increased frequency of CDR3 pairwise comparisons that differed by a distance of one or two for MT repertoires in the acute phase of infection compared to both SBP and unchallenged T_E_ repertoires, highlighting their greater repertoire similarity (**Figure 4B**).

### The Same Public Cluster Is Dominant in the Majority of Acute T-Effector Repertoires

**Figure 5:**
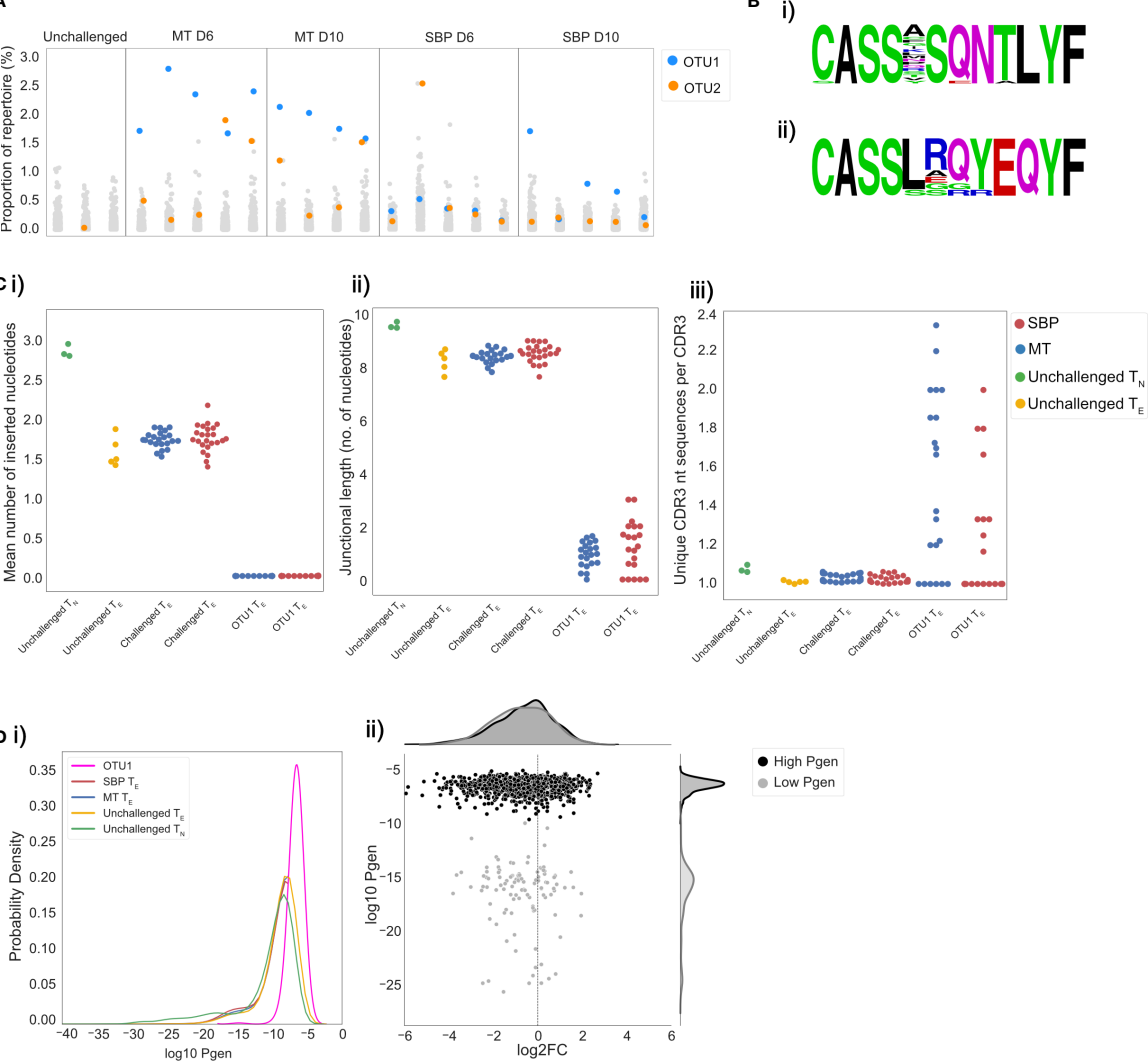
Acute mosquito-transmitted (MT) T_E_ repertoires are dominated by the same cluster of clones. Repertoires were clustered using a modified Swarm algorithm, to cluster CDR3 sequences within 1 amino-acid mismatch of each other, with identical V-gene usage. **(A)** Strip plots display the proportion of repertoire occupied by each cluster in individual repertoires for unchallenged T_N_ and MT and SBP T_E_ repertoires at day 6 and 10 post-infection. Each column is an individual repertoire, with each point representing a unique cluster. Cluster OTU1 is displayed in blue and OTU2 in orange. **(B)** Representative amino acid sequence logos of clusters OTU1 and OTU2. **(C)** OTU1 CDR3 sequences have hallmarks of a public response: (i) mean number of inserted random nucleotides in CDR3 sequences, (ii) mean number of nucleotides lying between V and J gene segment sequences and (iii) convergence (mean number of unique nucleotide sequences that encode a particular CDR3 sequence), for unchallenged T_N_ (green), unchallenged T_E_ repertoires (yellow), all MT T_E_ repertoires (blue) and all SBP T_E_ repertoires (red) and for cluster OTU1 in all MT and SBP T_E_ repertoires. **(D)** (i) Distributions of the generation probabilities (log10) of CDR3 nucleotide sequences for unchallenged T_N_ (green) and T_E_ repertoires (yellow), SBP (red) and MT (blue) T_E_ repertoires and CDR3 nucleotide sequences of cluster OTU1 (pink). (ii) Log2 fold change of clones present in unchallenged T_N_ repertoires versus challenged T_E_ repertoires. Each point represents an individual clone, and Pgen is separated in to high (>−10) (black) and low (<-10) (gray). Log2FC was calculated on UMI normalized data.

Given the increased connectivity in challenged repertoires, and the knowledge that TCRs recognizing the same antigen typically have a high global similarity to each other (38, 39), we clustered CDR3 sequences of individual repertoires within one amino acid mismatch of each other using a modified Swarm algorithm (26). This identified two clusters of highly similar CDR3 sequences, hereafter referred to as OTU1 and OTU2, that dominated T_E_ repertoires and were conserved across replicates in the acute phase of infection (**Figures 5A, B**). A near-identical cluster to OTU1 was also found to be expanded and dominant in a majority of challenged repertoires at day 7 and 11 post-infection (**Supplementary Figure 7B**), and both clusters were also observed in analyses of publicly available RNA-seq data sets for *P. chabaudi (AS)* and *P. chabaudi (CB)* (accession E-ERAD-221 and E-ERAD-289 respectively, **Supplementary Figure 7C**), showing a similar temporal pattern of expansion. We also applied the recently published Gliph2 algorithm (27) to our data. Gliph2 is designed to identify TCRs recognizing the same antigen by clustering sequences with enriched amino acid motifs in the high-contact-probability region of CDR3β (IMGT positions 107-116). It identified significant clusters that corresponded to both OTU1 and OTU2 in the acute phase of MT infections, as well several other clusters which were not as dominant nor as well-conserved in their response (**Supplementary Table 1**, **Supplementary Figure 7A**).

Given the conserved nature of OTU1 between individual mice, we hypothesized that the CDR3 sequences it contains would share similar properties with other known ‘public’ CDR3 sequences, defined simply as TCR clones shared by different individuals. Public TCR clones have been detected in numerous T-cell responses in multiple species, and although their functional significance remains unknown, they have been shown to be expanded in response to antigenic stimulation (40), viral infection (41, 42) and associated with regulatory self-immunity (43). In some previous studies, public sequences have been shown to have minimal alterations to germline V, D, and J gene sequences. In agreement with this, we found fewer recombination events in the CDR3 sequences in OTU1, with the mean number of randomly inserted nucleotides in the CDR3 sequences in these clusters significantly lower than that for CDR3 sequences in both challenged (t=−61.5, p<0.001) and unchallenged repertoires (t=−58.7, p<0.001) (**Figure 5Ci**). The mean number of nucleotides lying between the V and J gene segment sequences was also significantly lower (unchallenged: t=−22.1, p=0.002, challenged: t=−83.7, p<0.001) (**Figure 5Cii**). This cluster also showed a greater degree of convergent recombination – considered an important mechanism of public TCR generation (40, 44) - with a higher average number of unique CDR3 nucleotide sequences that code for the same CDR3 amino acid sequence (**Figure 5Ciii**) compared to CDR3 sequences in unchallenged (t=4.4, p<0.001) and challenged (t=5.3, p<0.001) repertoires. Consequently, CDR3 amino acid sequences in OTU1 had a higher probability of generation (Pgen) (29) than CDR3 sequences in unchallenged T_N_ repertoires and challenged repertoires (**Figure 5Di**). In a scenario of non-specific polyclonal expansion, a higher Pgen could indicate sharing and detection of this cluster incidentally due to higher abundance in the pre-selection pool, rather than as a result of common specificity (convergent selection) or function (45). However, the cluster was either not found or was present at a very low level in unchallenged T_N_ and T_E_ repertoires, and our use of UMI-corrected data for the repeat experiment confirmed that the CDR3s within this cluster are clonally expanded. Further, a large proportion of CDR3s with a similar high Pgen in unchallenged T_N_ populations, were either not present in T_E_ repertoires, or present but not expanded, demonstrating that the public cluster is likely to be pathogen-associated (**Figure 5Dii**).

To determine if the CDR3 sequences in OTU1 have been shown to expand in response to other antigenic stimuli in C57Bl/6 mice, we analyzed publicly available splenic CD4^+^ TCRβ repertoire data (43) from unchallenged mice and mice immunized with either OVA or CFA and OVA. Although detectable, the proportion of OTU1 did not differ between unchallenged and immunized mice, indicating the expansion seen is not simply an innate response to inflammation. TCR repertoire data was also extracted from publicly available whole blood RNA-seq data from C57Bl/6 mice challenged with a variety of pathogens; *Toxoplasma gondii*, influenza A virus, murine cytomegalovirus, respiratory syncytial virus, *Candida albicans*, *Listeria monocytogenes*, *Burkholderia pseudomallei*, and House dust mite allergen (30). Of the 9 pathogens examined at the peak of the murine response, only *P. chabaudi* elicited an expansion in TRBV3 gene usage (**Supplementary Figure 8**). As such, OTU1 was not found to be expanded by any of the other pathogens examined, supporting the probable specificity of this response.

### Splenic Memory and Re-Challenged Repertoires Are Predominantly Private Responses

**Figure 6:**
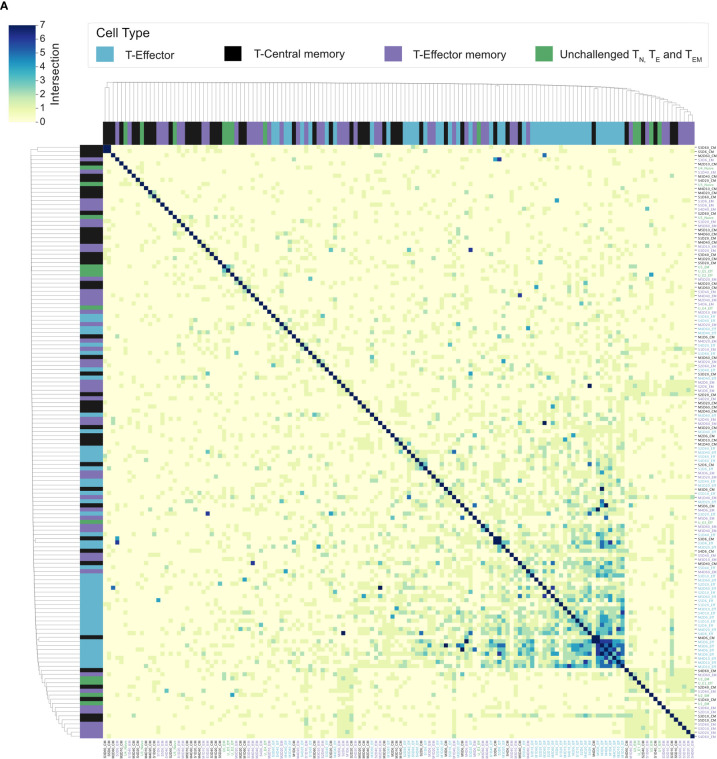
Splenic memory responses have little to no overlap: **(A)** Cluster map shows the pairwise T-cell receptor (TCR) repertoire overlap of the top 100 CDR3 sequences of individual repertoires, of challenged T_E_ (blue), T_CM_ (black), and T_EM_ (purple) repertoires, and unchallenged T_N_, T_E,_ and T_EM_ repertoires (green). The size of the pairwise intersection between each repertoire (number of shared CDR3 amino acid sequences) is displayed, with greatest overlap evident between acute T_E_ repertoires.

**Figure 7:**
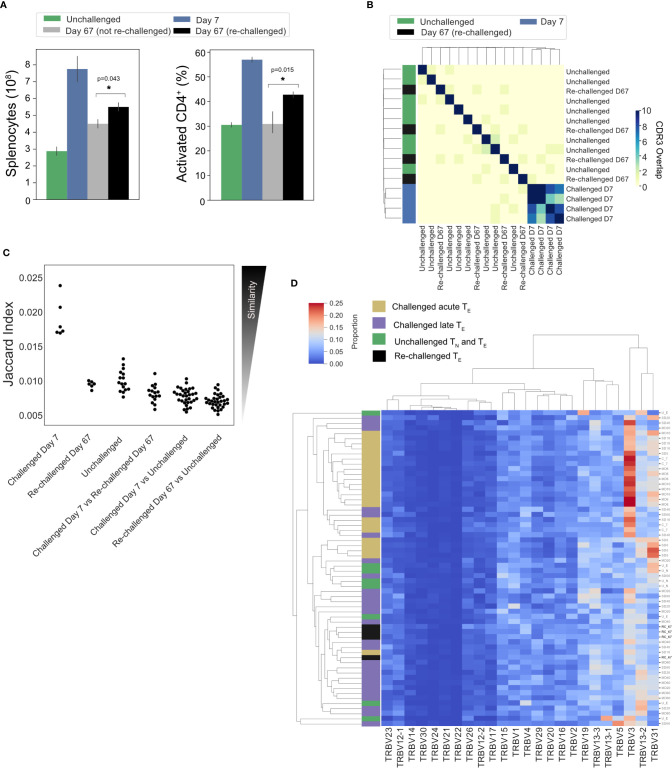
No conservation of response following homologous re-challenge: **(A)** Splenocyte numbers and frequency of activated CD4^+^T cells (CD44HI) of unchallenged mice (green), day 7 post-primary infection (blue), day 67 post primary infection (gray, not re-challenged) and 7 days following re-challenge (black, re-challenged day 67). **(B)** Clustermap depicts the pairwise overlap of the top 100 most abundant CDR3 amino acid sequences of repertoires from the re-challenge experiment. The size of the pairwise intersection between each repertoire (number of shared CDR3 amino acid sequences) is displayed. CDR3 overlap was calculated unweighted by clonotype frequency. **(C)** Jaccard (similarity) index of replicate repertoires (normalized by down-sampling to 5000 UMI) and **(D)** Clustermap displays the TRBV gene proportion of each repertoire for challenged acute T_E_ repertoires (gold), challenged late phase T_E_ repertoires (purple) and unchallenged T_N_ and T_E_ repertoires (green) from the first experiment, with re-challenged T_E_ repertoires (black) also included.

Splenic CD4^+^ T_EM_ and T_CM_ populations have been shown to expand following a *P. chabaudi* infection (8, 9, 20). To determine if a conserved splenic memory response was also generated, the TCRβ repertoires of splenic T_EM_ and T_CM_ populations from the first experiment were sequenced, for both infection types. All similarity and CDR3 overlap analyses were conducted unweighted, to avoid biases from effector clonal expansion. Little to no sharing was found between either the T_EM_ or T_CM_ replicate repertoires themselves for either infection type, nor between the T_EM_, T_CM_ and T_E_ populations (**Figure 6A**). Clustering the memory repertoires within one amino acid mismatch using the same modified Swarm algorithm (26) did not improve the similarity between memory replicates, indicating these responses are mostly ‘private’ to each individual at this sequencing depth. As a conserved response was not detected within memory populations, a homologous re-challenge infection was undertaken to determine if a similar expansion of TRBV3, as seen in primary infection, was observed upon secondary challenge. Mice were re-challenged at day 60 post-primary infection, and then sacrificed at day 67 (day 7 post re-challenge). Following re-challenge, mice did not develop a detectable parasitemia, but a splenic expansion of an activated effector population was evident, though not as marked as following primary challenge (**Figure 7A**). Although TRBV3 was again confirmed to be expanded 7 days after primary challenge, encoding 21.3% (± 3.6, 95% CI) compared to 6.0% (± 2.1, 95% CI) of unchallenged T_N_, no re-expansion of TRBV3 was detected following re-challenge. TRBV3 encoded 11.5% (± 0.4, 95% CI) of the re-challenged T_E_ repertoire, compared to 10.5% (± 1.7, 95% CI), for mice who had not undergone a secondary challenge, and re-challenged T_E_ TRBV gene repertoires clustered with later time-points rather than acute repertoires (**Figure 7D**). At the clonal level, re-challenged repertoires were found to be as dissimilar to each other as unchallenged repertoires (Mann Whitney U, p=0.29) (**Figure 7C**), with little to no sharing of the 100 most abundant CDR3 amino acid sequences (**Figure 7B**).

### Activated TRBV3^+^ Cells Have Diverse Transcriptional Phenotypes

**Figure 8:**
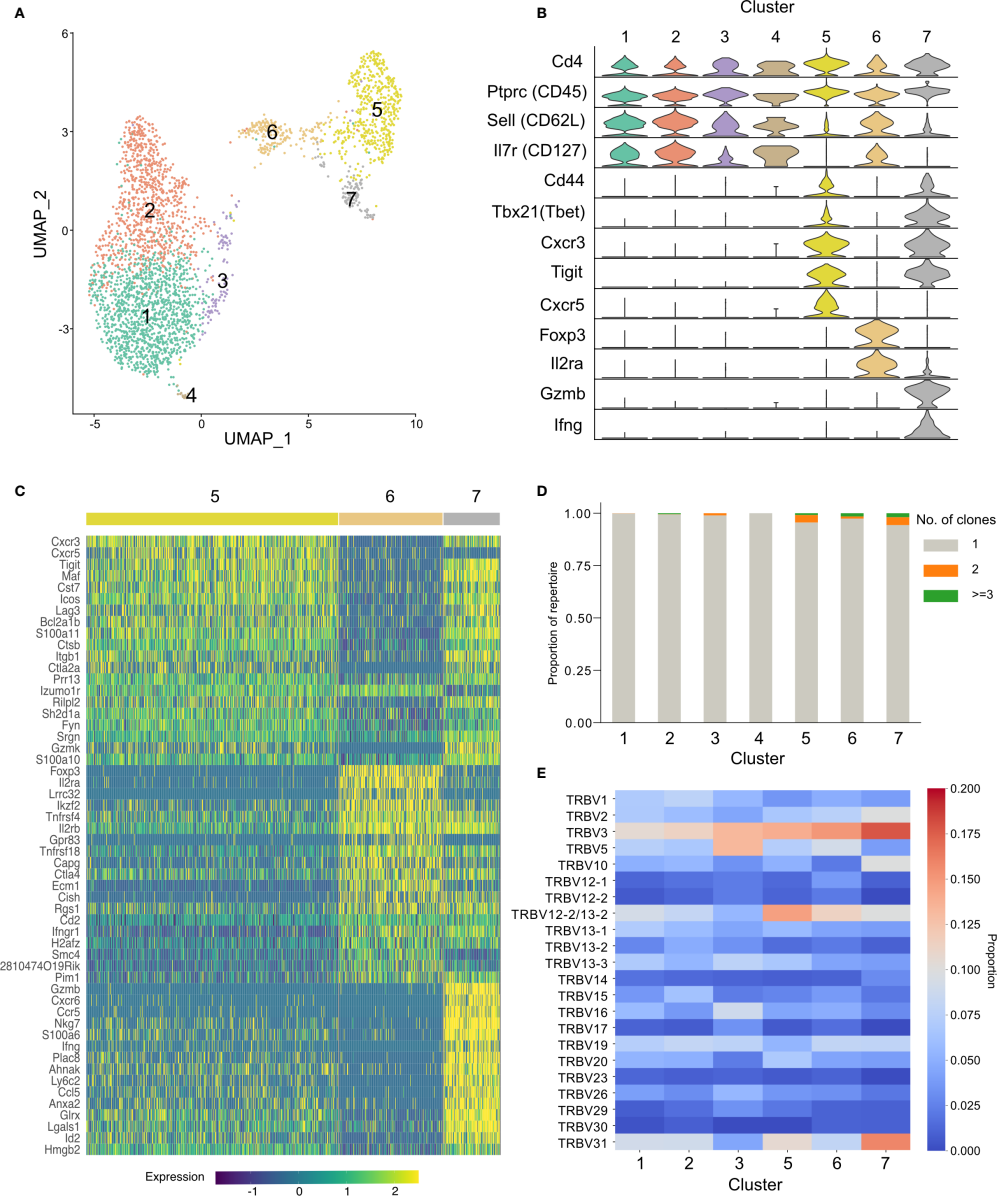
Activated TRBV3^+^ cells have diverse transcriptional phenotypes. Cellular transcriptional phenotypes and paired T-cell receptor (TCR) α and TCRβ sequences were captured using single-cell transcriptomics. **(A)** Clustering of CD4^+^ splenocytes from two challenged mice, at day 7 post-infection, according to transcriptional profile similarity. Plot shows individual cell transcriptomes represented in two dimensions by uniform manifold approximation and projection (UMAP). Each point depicts a cell, colored by Seurat cluster assignment as determined from gene expression profiles. **(B)** Distributions of normalized expression level of major canonical T-cell markers for each cell cluster, displayed as violin plots. **(C)** Heatmap showing the expression levels of the top 20 differentially expressed genes that define clusters 5, 6, and 7. **(D)** Stacked bar plot showing the proportion of each cluster that is clonally expanded (>1 cell with identical paired TCRα and TCRβ aa sequences). Repertoires of all clusters were highly polyclonal, with a minor increase in frequency of expanded clones in activated clusters. **(E)** Heatmap displays proportional TRBV gene usage per cluster TCR repertoire.

To further our understanding of the phenotype of the conserved TRBV3 response observed during the acute phase of a *P. chabaudi* infection, we undertook single-cell RNA sequencing of FACS sorted CD3^+^ CD4^+^ splenocytes from two mice at day 7 post-infection with MT parasites. We sequenced these cells using the 10x Genomics Chromium platform, using the V(D)J enrichment protocol to obtain paired α/β TCR data for each cell. After quality control steps, we obtained expression profiles for 1491 and 1485 CD4^+^ T cells from each mouse respectively (2976 total). Following dimensionality reduction by principal component analysis (PCA), we undertook graph-based clustering (32) and visualized resulting populations using uniform manifold approximation and projection (UMAP) (**Figure 8A**). We identified seven discrete transcriptional clusters, with cells from both mice evenly distributed across all dominant clusters (**Supplementary Figure 9**). Four of these clusters, denoted as clusters 1, 2, 3, and 4, were classified overall as naïve on the basis of canonical markers (*Sell*, *Il7r*) (**Figure 8B**, **Supplementary Figure 10**). Differential expression between all seven clusters indicated that clusters 2 and 3 were distinguishable by markers indicative of early T-cell activation, including *cd69*, *ltm2a*, *Zfp36* and *ler2* for cluster 3, and type I interferon (IFN) response genes (*Gbp2*, *Ifit1*, *Ifit3*, *Isg15*, and *Socs1*) (46) for cluster 2 (**Supplementary Figure 11**), while cluster 1 expressed a more definitive naïve phenotype (*lef1*, *Ccr7*). We also identified three clusters discrete from the naïve group: cluster 6 showed a typical Treg transcriptional phenotype with expression of *Foxp3* and *Il2ra*, while clusters 5 and 7 were both identified as activated effector populations (*Sell-*, *Il7r-*). Cluster 7 expressed genes associated with a pro-inflammatory T_H_1 signature, including *Tbx21, Ifng, Gzmb, Cxcr6, Ccl5, NKg7*, and *Bhlhe40* (47). In contrast, cluster 5 was *Ifng-* and appeared to be a more diverse helper population, which predominantly included cells expressing genes associated with a T_FH_ phenotype (*Cxcr5*, *Icos, Bcl6, Il21, Pdcd1)* as well as those with a T_H_2 phenotype (*Gata3, Ccr4*) (**Figure 8C**). Utilizing the TCR data, we confirmed that the effector response is highly polyclonal: of the 581 effector cells present in clusters 5 and 7, only 3.6% (21/581) are clonally expanded (>1 cell with identical paired TCRα and TCRβ aa sequence) (**Figure 8D**), and no clone had more than three copies present. We again confirmed that TRBV3 is the most dominant TRBV gene encoding the TCRβ chain of activated effector populations, and show that out of all the clusters, cluster 7 had the highest proportion of TRBV3 (**Figure 8E**). Cells that were TRBV3^+^ did not however form a distinct cluster and instead displayed diverse phenotypes across all clusters. No TRAV gene was over-represented in a particular cluster (**Supplementary Figure 9B**). Of the clonotypes previously identified in the TCRβ bulk data in OTU1 and OTU2, two were present in the single-cell data set, both within the predominantly T_FH_ cluster 5. TCRβ nucleotide sequences displayed diverse probabilities of generation across all clusters (**Supplementary Figure 12**).

## Discussion

CD4^+^ T-cells play a critical role in the immune response against the pathological blood-stage of malaria [reviewed in (6, 7)]. However there is a lack of deep mechanistic understanding regarding the development of T-cell mediated immunity against *Plasmodium* (7). To our knowledge, this is the first study to use bulk TCR deep-sequencing to examine the composition of CD4^+^ T-cell repertoires induced by a *Plasmodium* infection. We found that in both the MT and SBP infection models, which show differential control of parasite growth and degrees of immunopathology (8, 16), T_E_ repertoires elicited upon infection are highly diverse and polyclonal. Despite the 10-fold increase in splenic T_E_ cell numbers, the V/J gene usage frequencies of acute challenged T_E_ repertoires are strongly positively correlated with that of unchallenged T_N_ repertoires, indicating a highly polyclonal broad expansion of the naïve repertoire, consistent with the massive cellular expansion seen. This polyclonality is confirmed in our single-cell RNA-seq data. The degree of correlation between VJ usage frequencies likely indicates a degree of non-specific expansion, although a highly heterogeneous response to the vast number of potential antigens expressed by the parasite, argued as a potential cause for the preponderance seen in *P. falciparum* IgG responses (48), cannot be ruled out. However, within this highly polyclonal effector proliferation, a strong and oligo-clonal conserved response is observed in the bulk TCR data following a first infection. Here, repertoires are skewed to TRBV3 gene usage, have a higher degree of clonal sharing, and show increased amino acid sequence similarity of the CDR3 region between dominant clones. Thus, we demonstrate that despite the antigenic complexity of *P. chabaudi*, T_E_ repertoires bear the hallmarks of a specific response (38, 39), mirroring that observed with less antigenically diverse organisms (49–51). This conserved response is evident in both infection models, although it is delayed and less marked in mice infected with SBP parasites, and does not become evident until parasitemia has peaked. Adaptive immune responses of lower magnitude have previously been documented in SBP infections compared to MT (8) and while the precise reasons for this difference remain speculative and represent a complex scenario, the fact that the conserved response does eventually become evident suggests that the drivers of this response are likely to be common to both MT and SBP parasites despite apparent distinct kinetics in the TRBV3 response. Sequencing of total parasite RNA for both MT and SBP parasites undertaken by Spence et al. (8), indicated the parasites’ *cir* gene family - believed to encode a large set of variable antigens displayed on parasitized erythrocytes -was differentially expressed between the two infection models. MT parasites upregulated expression of many genes within this family equally, re-setting broad expansion of the antigenic repertoire, while SBP selected for a limited but dominant *cir* expression. Our results are therefore inconsistent with proteins encoded by *cir* genes driving the detected conserved response, which would instead have been expected to result in a more dominant response in SBP infections. We hypothesize that the differences observed in the timing and magnitude of the TRBV3-restricted shared response seen between the two infection models are a result of the systemic inflammation induced by SBP parasites (8) disrupting or delaying the formation of an appropriate T-cell response. Future work to determine MHC-presentation pathway and ligands or peptides involved is ultimately required to determine what specific parasite epitope elicits the conserved response.

The single cell RNA-seq data confirms the dominance of TRBV3 in activated effector populations and reveals that TRBV3^+^ cells in the acute phase of a first infection have diverse phenotypes. We therefore show that the conserved response seen does not represent a discrete innate cell population but is instead part of an adaptive immune process against the parasite. The transcriptional phenotypes of activated effector cells are in agreement with those previously observed in the acute phase of a *P. chabaudi* infection (47) and indicate a large cluster of predominantly follicular helper cells as well as a distinct *Ifng*^+^ T_H_1 population. Both types of response have been shown to arise simultaneously during the acute phase (47) and to be essential in controlling blood-stage *P. chabaudi*; T_H_1 responses are required for initial control of acute parasitemia (52, 53) and T_FH_ are crucial for generating antibody-mediated immunity and controlling chronic infection (54, 55). The presence of OTU1 and OTU2 in a predominantly T_FH_ cluster, suggests that they may play a role in guiding the developing humoral response against the parasite. A single naïve CD4^+^ T-cell has been demonstrated to be able to give rise to clones with different cell fates (47) so, as only one of each clonotype was captured with the single cell sequencing depth used, we cannot firmly conclude that the transcriptional phenotypes identified here would broadly reflect the conserved TCRs identified in the bulk data. Nevertheless, the TCR of a cell has been shown to impart a strong preference for either a T_H_1 or a T_FH_ fate, with longer dwell time between peptide-MHC : TCR biasing toward T_FH_ and GC-T_FH_ responses (56). Therefore, if driven by the same epitope, we would expect the conserved response to have a similar phenotype (57).

Public TCR sequences are shared between multiple individuals either due to biases in V(D)J recombination, and/or convergent selection by a common antigen (45, 49). The CDR3 amino acid sequences in the most dominant and conserved cluster detected (OTU1) had similar features to other previously observed public TCR responses. These include a reduced number of nucleotide addition and deletion events during VDJ recombination and a greater degree of recombinant convergence (different nucleotide sequences encoding the same amino acid CDR3 sequence). Such recombinational biases during T-cell development mean these TCRs will have a higher probability of generation (PGen) during VDJ recombination, and are therefore more likely to be present in a naïve pool. However, the degree of clonal expansion observed in OTU1 sequences was greater than many other sequences of equal or higher PGen, indicating that these clones were truly expanded and not simply found to be of high frequency as a result of recombinational biases. We also show that PGen does not determine T-cell fate, in agreement with Sethna et al. (29) who demonstrated that the ability of a TCR to respond to a particular epitope, was not strongly correlated with its generation probability. It has been hypothesized that public TCR responses may provide rapid cross-reactive immunity (58) to cope with diverse antigenic challenge, allowing time for more specific private responses to develop (40, 59). Thus, during a *P. chabaudi infection*, a public response that is mobilized rapidly due to high Pgen and/or a higher chance of positive selection if cross-reactive, may act as a first line of defense against the parasite before more specific responses become effective. In agreement with this, OTU1 appears to be temporally associated with enhanced control of parasitaemia. It arises earlier and is more dominant in MT infections compared with SBP, where rapid parasite growth is observed alongside a delayed and less marked conserved response. Mice infected with MT parasites also show reduced disease severity (8). Despite these positive associations, whether this conserved response is truly beneficial to the host, remains unknown. There are reports of public TCR responses being implicated in self-related immunity (43), and in *P. berghei*, the presence of conserved pathogenic CD8^+^ T-cells has been used to predict cerebral malaria (11).

No expanded conserved response was evident in memory populations at the timepoints tested. It is known that precursors of memory T-cells derive from an earlier T_E_ population (CD127^-^ CD62L^hi^) that precedes the terminally differentiated CD62L^-^ CD127^-^ T_E_ population captured by our T_E_ gating strategy (60). While that precursor population successfully expands to dominate the T_E_ repertoire, since memory T populations preferentially favor TCRs with high avidity, it is possible that the public TCR clones identified may lack sufficient avidity required to enter the memory pool (61). A low level of T_M_ repertoire overlap indicates that splenic memory responses are diverse and mostly private to each individual. Similarly, in the re-challenged T_E_ compartment, we did not detect an increase in TRBV3 compared to later time points that had not undergone re-infection, and at the clonal level responses had as little overlap between replicates as unchallenged repertoires. This does not imply lack of a memory/primed response; mice are protected from re-infection and have previously been shown to have primed secondary responses (9). Heterologous responses could be due to T-cells more specific for the parasite than the TRBV3+ public response surviving the contraction phase/entering memory to be able to respond. Diverse or ‘private’ responses have previously been seen with other complex pathogens such as *mycobacteria* (62, 63) and would not be unexpected given the antigenic complexity of *Plasmodium*. However, primed private secondary responses cannot be confirmed without experiments that sample the same individuals longitudinally over the course of a primary and secondary infection, and this hypothesis therefore remains untested and an avenue for future investigation.

These results also do not exclude the possibility that a low-level re-expansion of TRBV3 does occur upon re-challenge, given only a small increase in the number of activated cells was observed. This may not have constituted a large enough proportion of the repertoire, given the number of animals used in re-infection, to detect subtle differences in re-challenged T_E_ V-gene usage. This is supported by work done by Opata et al. (9), who demonstrated that T-cells specific to *P. chabaudi* MSP-1 that survive the contraction phase, do not re-expand upon secondary challenge due to increased apoptosis. Such clones that are responding but do not accumulate/re-expand would be difficult to discern from baseline controls using bulk repertoire sequencing techniques. Future re-infection studies may benefit from including single-cell transcriptomics to help differentiate such responding cells based on phenotype.

To have found a near-identical expanded T_E_ cluster in independent repeat experiments, and in publicly available *P. chabaudi* (AS and CB) RNA-seq data sets, demonstrates the public TCR signature is consistently expanded in multiple individuals in response to a first *Plasmodium chabaudi* infection. TRBV3 was not expanded at peak infection for any of the other eight pathogens examined and searches of annotated TCR sequence databases have not revealed any other known specificities for any of the CDR3 amino acid sequences in OTU1 or OTU2 (50, 64). TCR repertoire and RNA-seq data sets following infection with other *Plasmodium* species are currently unavailable, but as these are generated, the specificity of this response to the parasite and whether a similar response is elicited in a first infection by other *Plasmodium* species including *P. falciparum* will become evident.

Future studies are required to determine whether the conserved response plays a critical role in protection during a first infection. MT infections develop chronic recrudescing infection, so the response does not fully clear infection, although it is temporally associated with control of parasite growth and reduced disease severity (8). Whether entirely favorable to the host or not, we hypothesize the response allows time for mechanisms that govern the formation of more specific private responses and subsequent immunity to the parasite to develop. Mice are known to develop highly effective strain-specific anti-parasite immunity after a single malaria episode, while this takes years to develop in people. Therefore, if some degree of first line protection were shown, such a response could be a novel target to promote in malaria naïve individuals through vaccination, providing initial cover while more specific but slower private responses develop, conveying higher levels of protection. Even if only partially protective, the response is conserved between individuals and receptor sequences have a high probability of generation. Targeting these is predicted therefore to increase vaccine success rate (29, 65).

**Figure 9:**
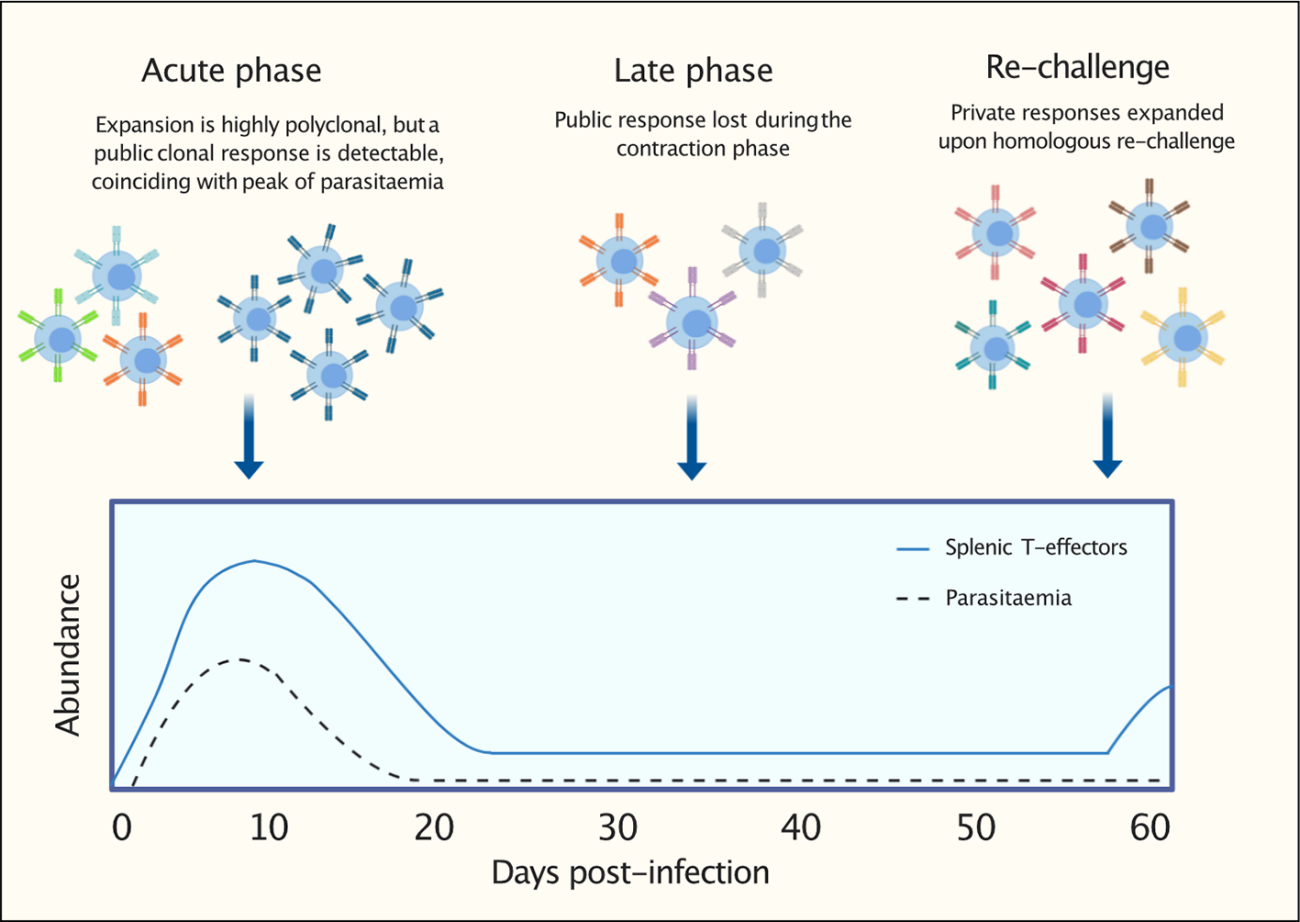
Graphical representation to summarize findings: following a first *P. chabaudi* infection, within a highly polyclonal T-effector expansion, a public T-cell receptor (TCR) β clonal response encoded by TRBV3 is detectable. This conserved response contracts during the late phase of infection and is not re-expanded upon homologous re-challenge. This response is consistently found to be a hallmark of a first *P. chabaudi* infection.

In summary, we have demonstrated that a conserved TCRβ signature encoded by TRBV3 is consistently expanded in response to a first *Plasmodium chabaudi* infection (**Figure 9**). In contrast, memory formation and re-challenged repertoires appear to be more private responses to the individual. Understanding the antigenic drivers and contribution to protection (or pathogenesis) of this conserved signature, that is consistently a hallmark of a first infection, is ultimately required to determine if it should be promoted or mitigated for malaria therapeutic purposes.

## Data Availability Statement

The datasets presented in this study can be found in online repositories. The names of the repository/repositories and accession number(s) can be found below: https://www.ebi.ac.uk/ena, PRJEB40867 https://www.ebi.ac.uk/arrayexpress/, E-MTAB-9691.

## Ethics Statements

The animal study was reviewed and approved by the Ethical Review Body of the University of Edinburgh.

## Author Contributions

NS, GC, and CS designed the project, analyzed the data, and wrote the manuscript. Experimental work was carried out by NS, GC, WN, JM, PS, and JT. All authors reviewed the manuscript. All authors contributed to the article and approved the submitted version.

## Funding

This work was made possible by a studentship from the Wellcome Trust to NS (204511/Z/16/A) and Wellcome Trust ISSF funding to GC.

## Conflict of Interest

The authors declare that the research was conducted in the absence of any commercial or financial relationships that could be construed as a potential conflict of interest.
