# Supplementary material for: A Conserved TCRβ Signature Dominates a Highly Polyclonal T-Cell Expansion During the Acute Phase of a Murine Malaria Infection
Source: Front Immunol. 2020 Nov 23;11:587756. doi: 10.3389/fimmu.2020.587756 (PMC7719809; doi:10.3389/fimmu.2020.587756)
Supplement: Supplementary file 1 [file Image_1.pdf]

Supplementary Figure 1

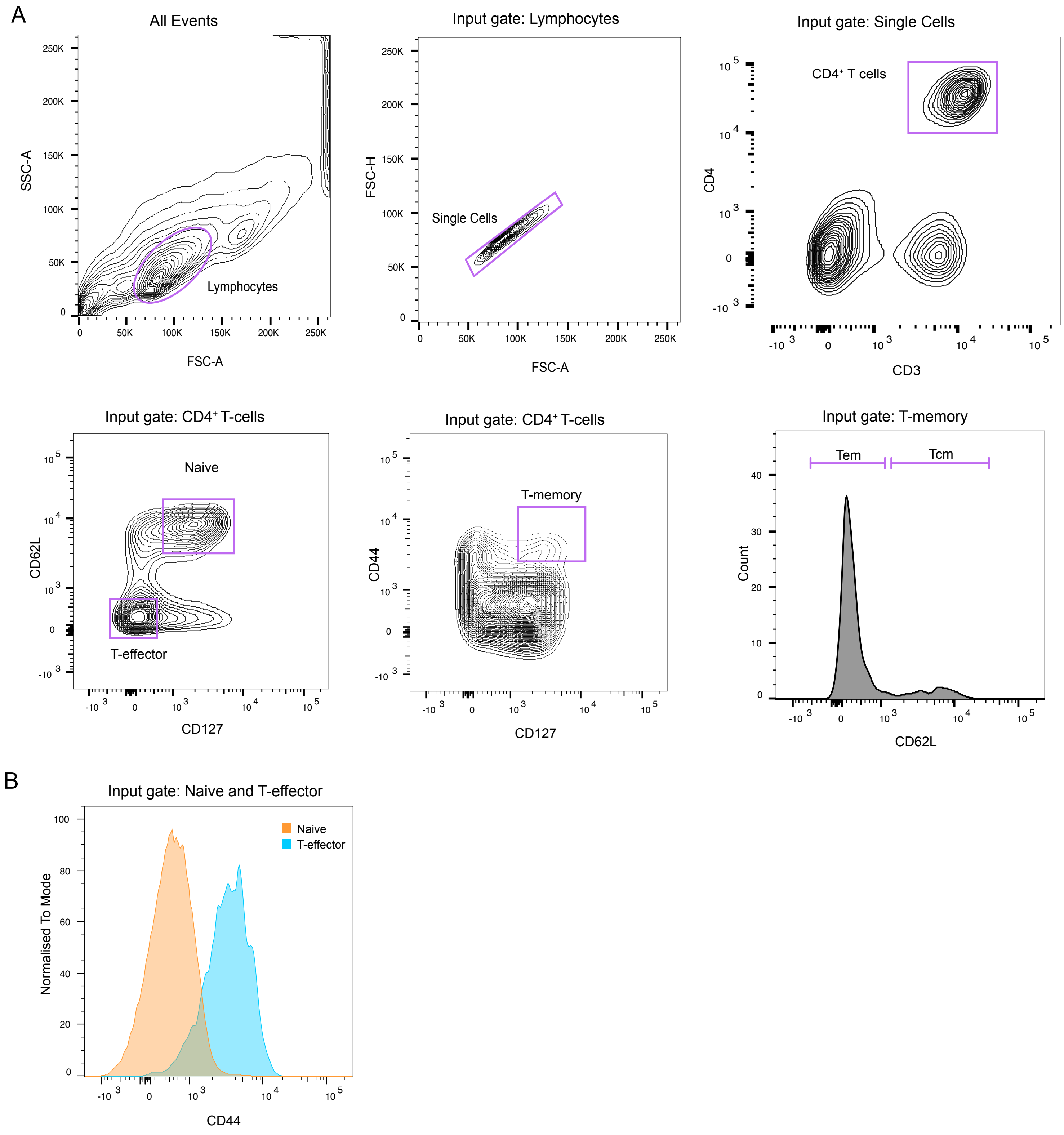

Supplementary Figure 1: A) Gating strategy for T-cell populations, illustrated from a representative mouse infected with SBP *P. chabaudi (AS)* at day 6 post-infection. B) Histogram showing CD44 level of Naive and T-effector T-cell populations.

Supplementary Figure 2

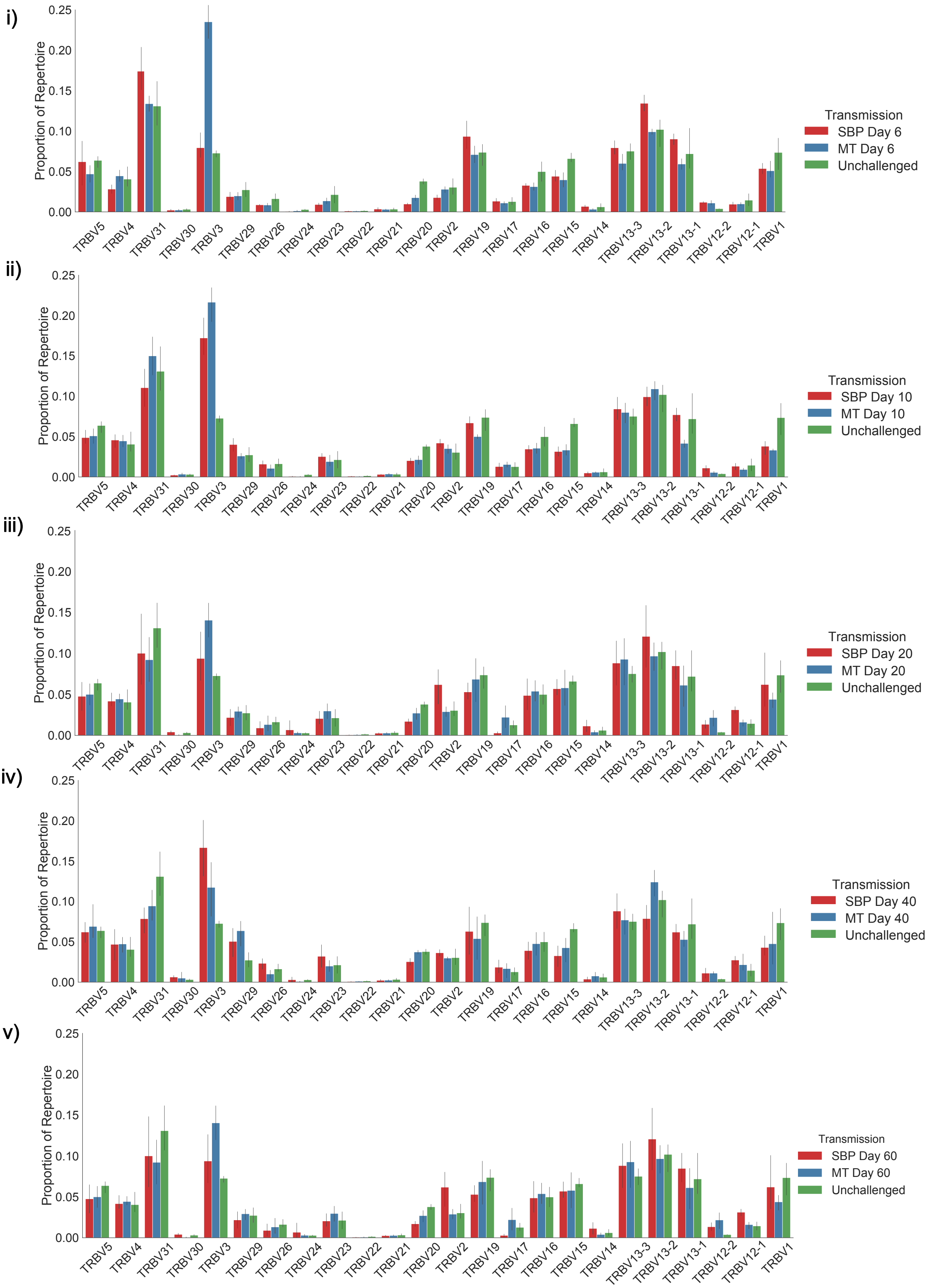

Supplementary Figure 2: Proportion of TRBV-gene usage in unchallenged T_N repertoires (green), and T_E repertoires of mice infected with SBP parasites (red) or recently MT parasites (blue) at days 6 (i), 10 (ii), 20 (iii), 40 (iv) and 60 (v) days post-infection.

Supplementary Figure 3

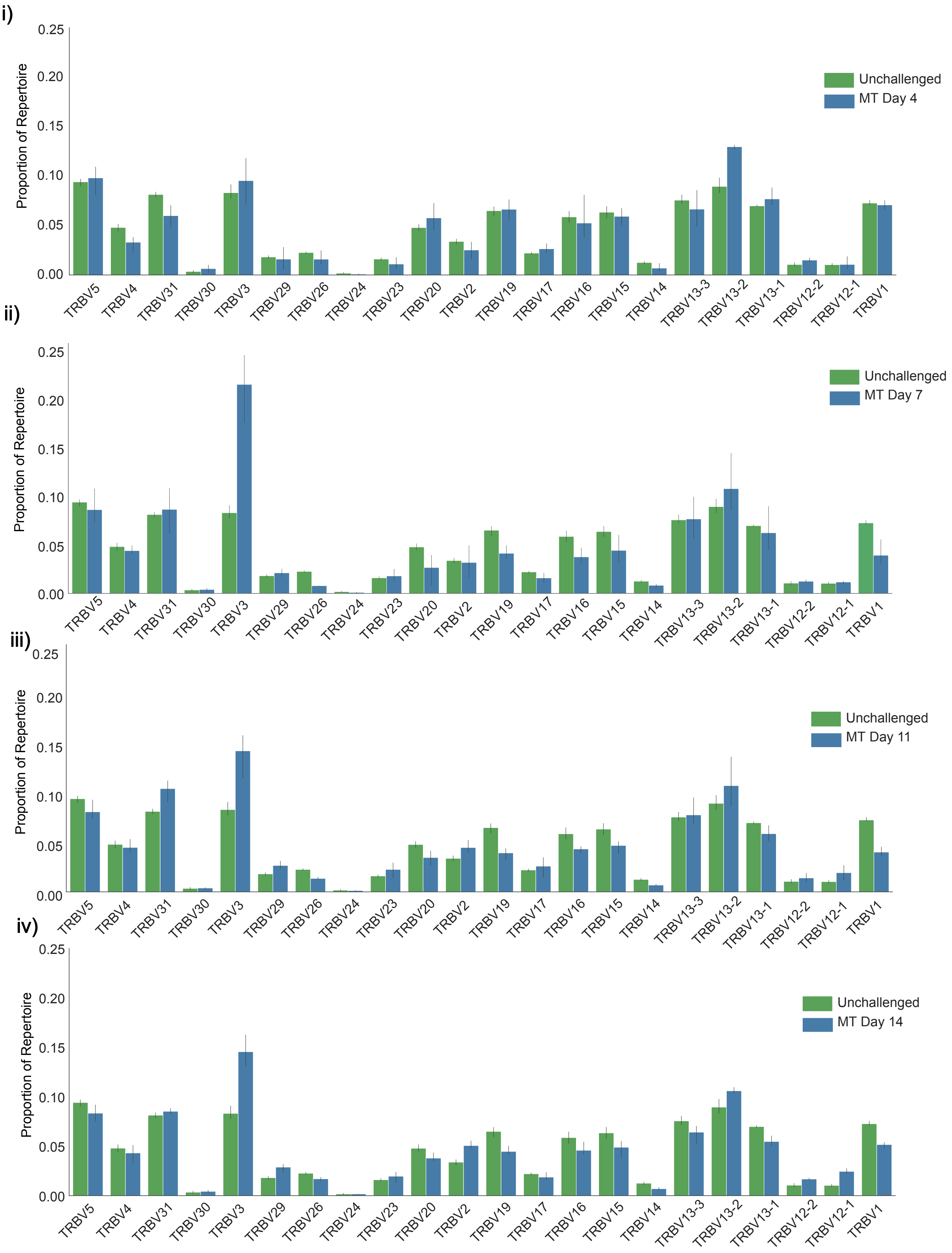

Supplementary Figure 3: Proportion of TRBV-gene usage in unchallenged T_N repertoires (green) and T_E repertoires of mice infected with recently MT parasites (blue) in a second independent experiment at days 4(i), 7 (ii), 11 (iii) and 14 (iv) post-infection.

Supplementary Figure 4

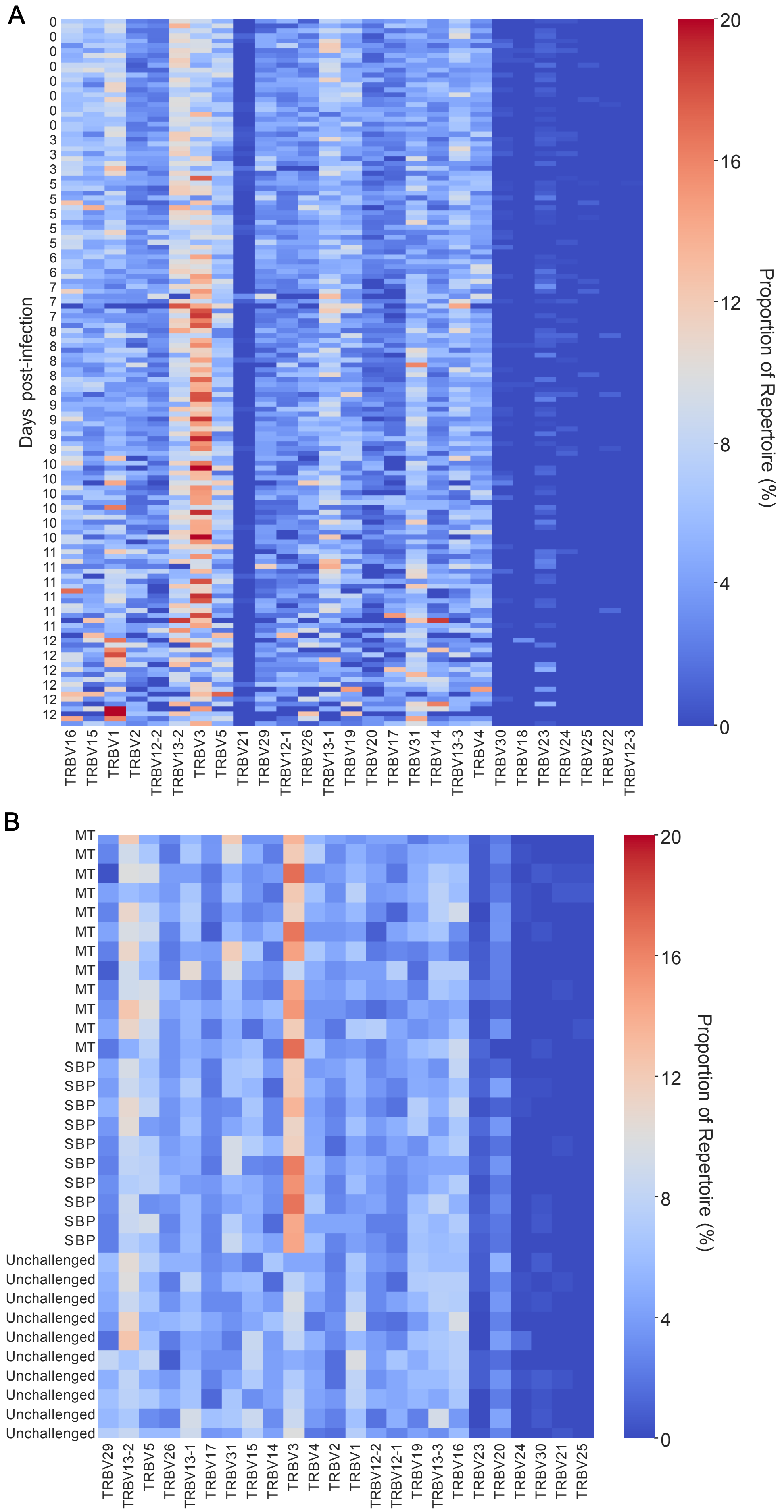

Supplementary Figure 4: Heatmaps show proportion of TRBV-gene usage in unchallenged and challenged mice unsorted splenic TCRβ repertoires from C57BL/6 mice infected with bloodstage A) *P. chabaudi (AS)* and B) *P. chabaudi (CB)*. TCRβ repertoires were reconstructed from publicly available RNA-seq data sets. Each column represents a unique TRBV gene, and each row is an individual replicate mouse. No time-point data is available for (B).

Supplementary Figure 5

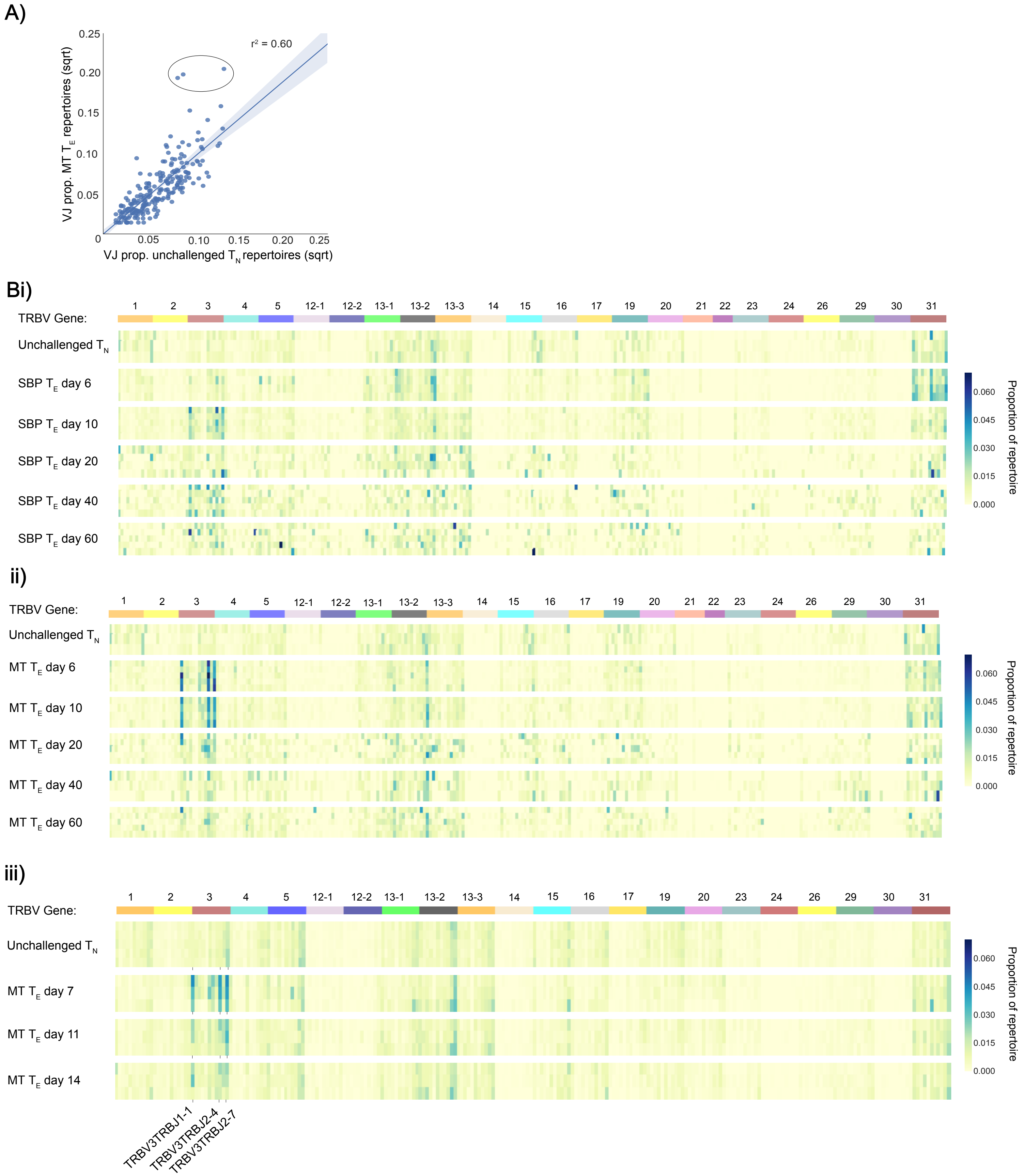

Supplementary Figure 5: Mean proportion of each V/J allelic combination in unchallenged T_N repertoires versus challenged T_E repertoires at days 7 (i) post-infection for mice infected with recently MT parasites in a second independent experiment. B) Heatmap depicts proportion of each V/J allelic combination (columns) for individual replicate mouse (rows) for (i) unchallenged T_N repertoires and acute T_E SBP repertoires and (ii) unchallenged T_N repertoires and acute T_E MT repertoires and (iii) unchallenged T_N repertoires and acute T_E MT repertoires from a second independent experiment.

Supplementary Figure 6

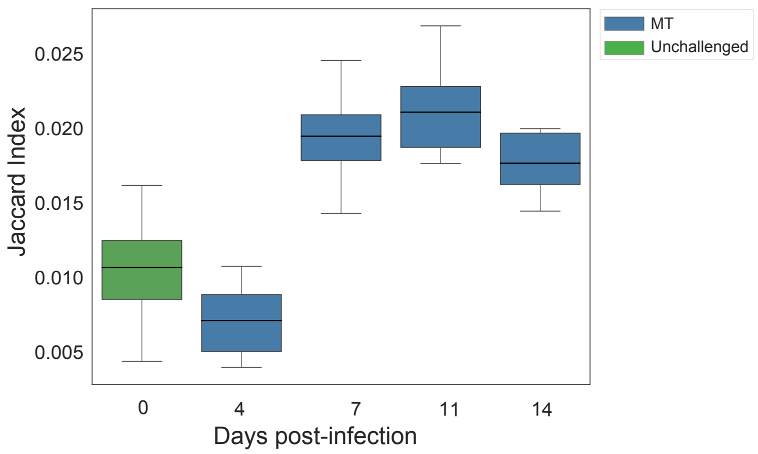

Supplementary Figure 6: Jaccard similarity index of unchallenged T_N repertoires (green) and T_E repertoires of mice infected with recently MT parasites (blue) in a second independent experiment at days 4, 7, 11 and 14 post-infection. Data was normalised by down-sampling to 5000 UMI.

Supplementary Figure 7

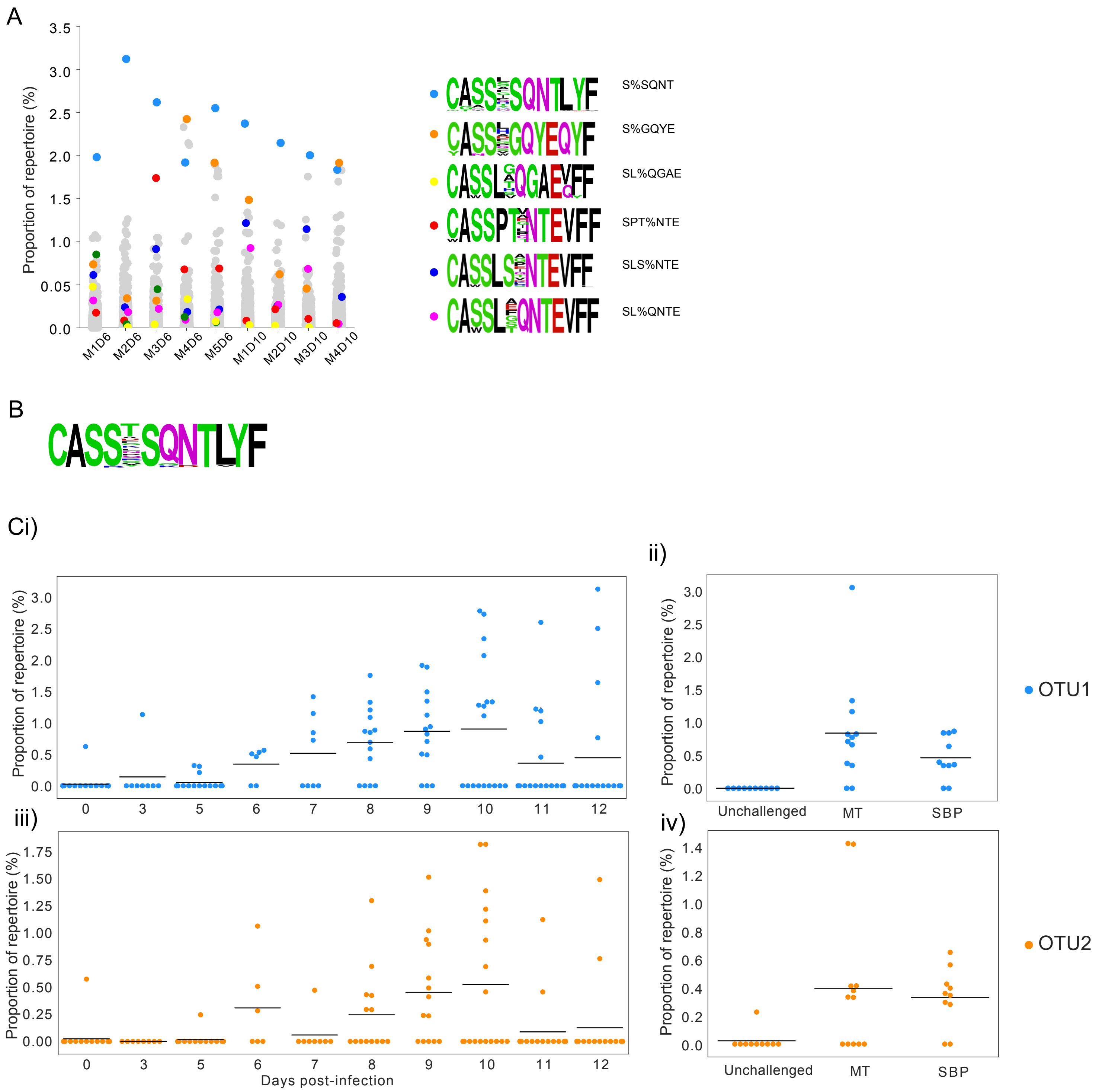

Supplementary Figure 7: Strip plots indicate proportion of individual repertoires taken up by clusters identified as significantly enriched using GLIPH2 (results in Supplementary Table 1), with representative sequence logos for each cluster. B) Representative amino acid sequence logo of the most dominant cluster detected (OTU1) from a second independent experiment. C) Proportion of CDR3s in cluster OTU1 (blue) and OTU2 (orange) found in unsorted splenic TCRβ repertoires reconstructed from publicly available RNA-seq data for i) and iii) *P. chabaudi (AS),* and for ii) and iv) *P. chabaudi (CB).* No time point data is available for the *P.chabaudi* (CB) dataset. Black horizontal line indicates mean, and each point represents an individual mouse.

Supplementary Figure 8

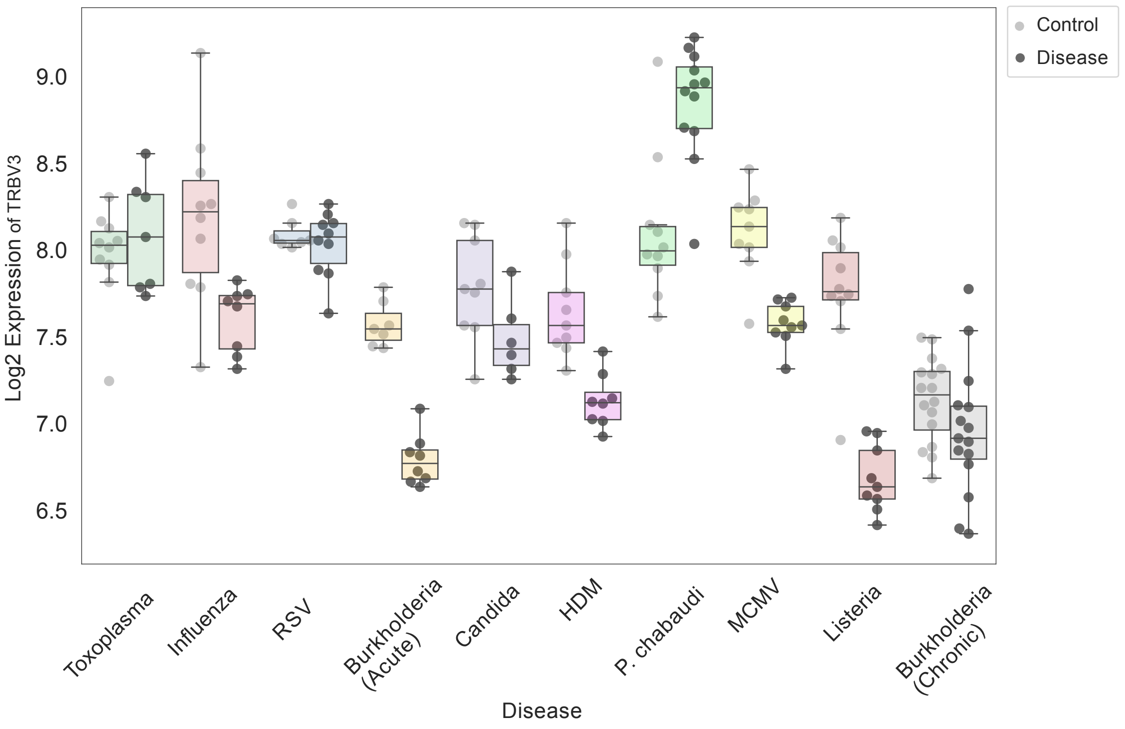

Supplementary Figure 8: Log2 expression values (normalised RNA-seq counts) of TRBV3 from publicly available whole blood RNA-seq data from C57Bl/6 mice, taken at peak of murine response for each disease. Data is paired as unchallenged controls (light grey circles) and challenged (black circles).

Supplementary Table 1: Summary results for GLIPH2 analysis. Filter criteria used: CDR3 sequence present in at least 75% of replicate group; vb_score<=0.05; length_score <=0.05; expansion_score <=0.05; Fisher_score <=0.001

Infection type	Time point	No. of clusters meeting criteria	Cluster_ID	Pattern	Final_score	
MT	D6	5	14	S%SQNT	8.50E-14	OTU1
			18	SPT%NTE	3.10E-13	
			10	SLS%NTE	4.60E-13	
			4	SLTQ	4.60E-12	
			7	SL%QGAE	8.50E-12	
MT	D10	4	21	S%SQNT	7.70E-14	OTU1
			32	S%GQYE	4.40E-13	OTU2
			5	SLS%NTE	1.70E-12	
			23	SL%QNTE	6.10E-12	
MT	D20	0				
MT	D40	0				
MT	D60	0				
SBP	D6	0				
SBP	D10	1	16	S%GTTSAET	1.10E-09	
SBP	D20	0				
SBP	D40	0				
SBP	D60	0				
