# Supplementary material for: A Conserved TCRβ Signature Dominates a Highly Polyclonal T-Cell Expansion During the Acute Phase of a Murine Malaria Infection
Source: Front Immunol. 2020 Nov 23;11:587756. doi: 10.3389/fimmu.2020.587756 (PMC7719809; doi:10.3389/fimmu.2020.587756)
Supplement: Supplementary file 2 [file Image_2.pdf]

Supplementary Figure 9

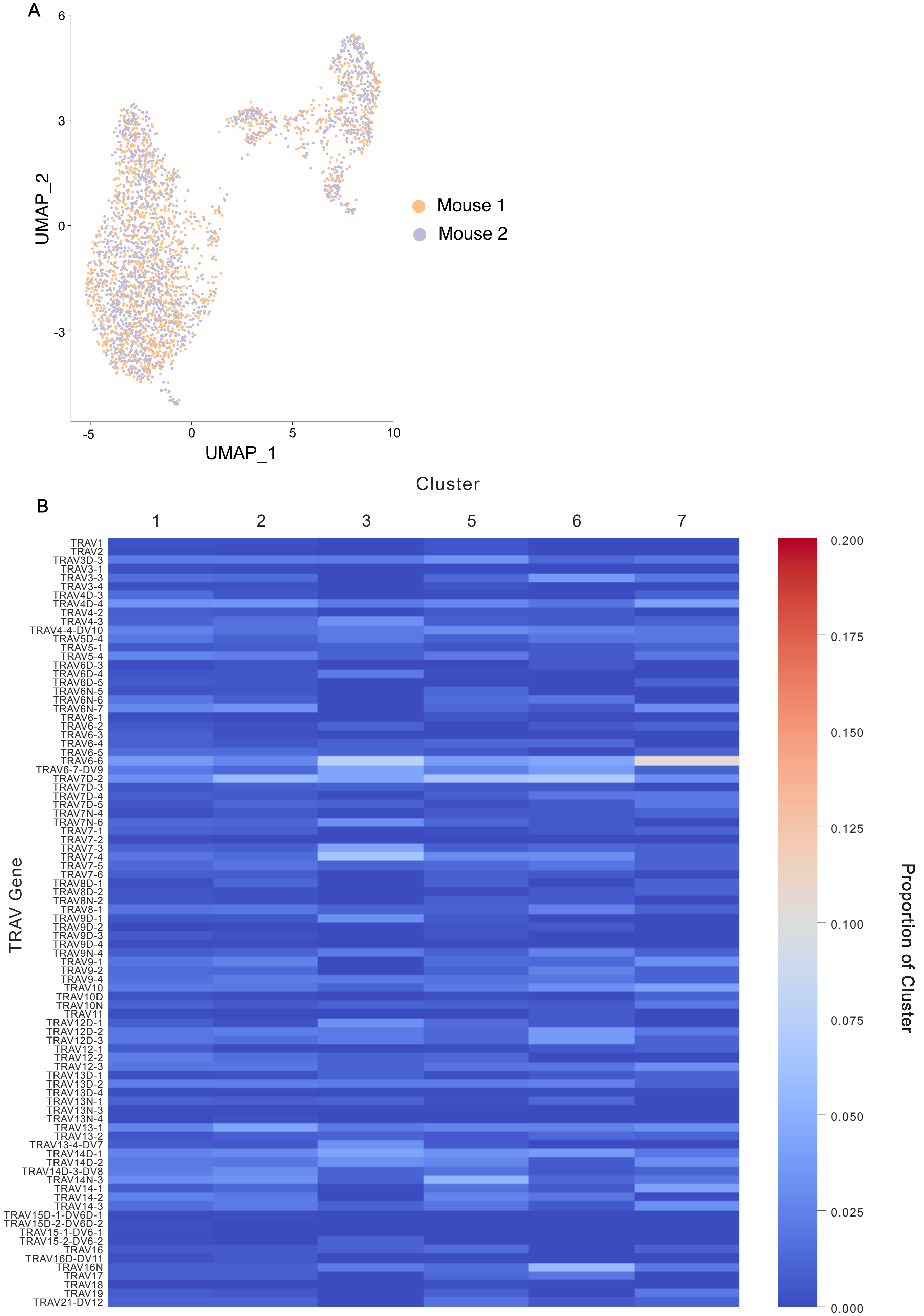

Supplementary Figure 9: A) Two-dimensional UMAP visualization of CD4⁺ splenocytes from 2 challenged mice, at day 7 post-infection, as per Seurat cluster assignment, with cells coloured by individual; cell positions are as per UMAP in Figure 8A. B) Heatmap displays proportion of TRAV gene per cluster TCR repertoire.

Supplementary Figure 10

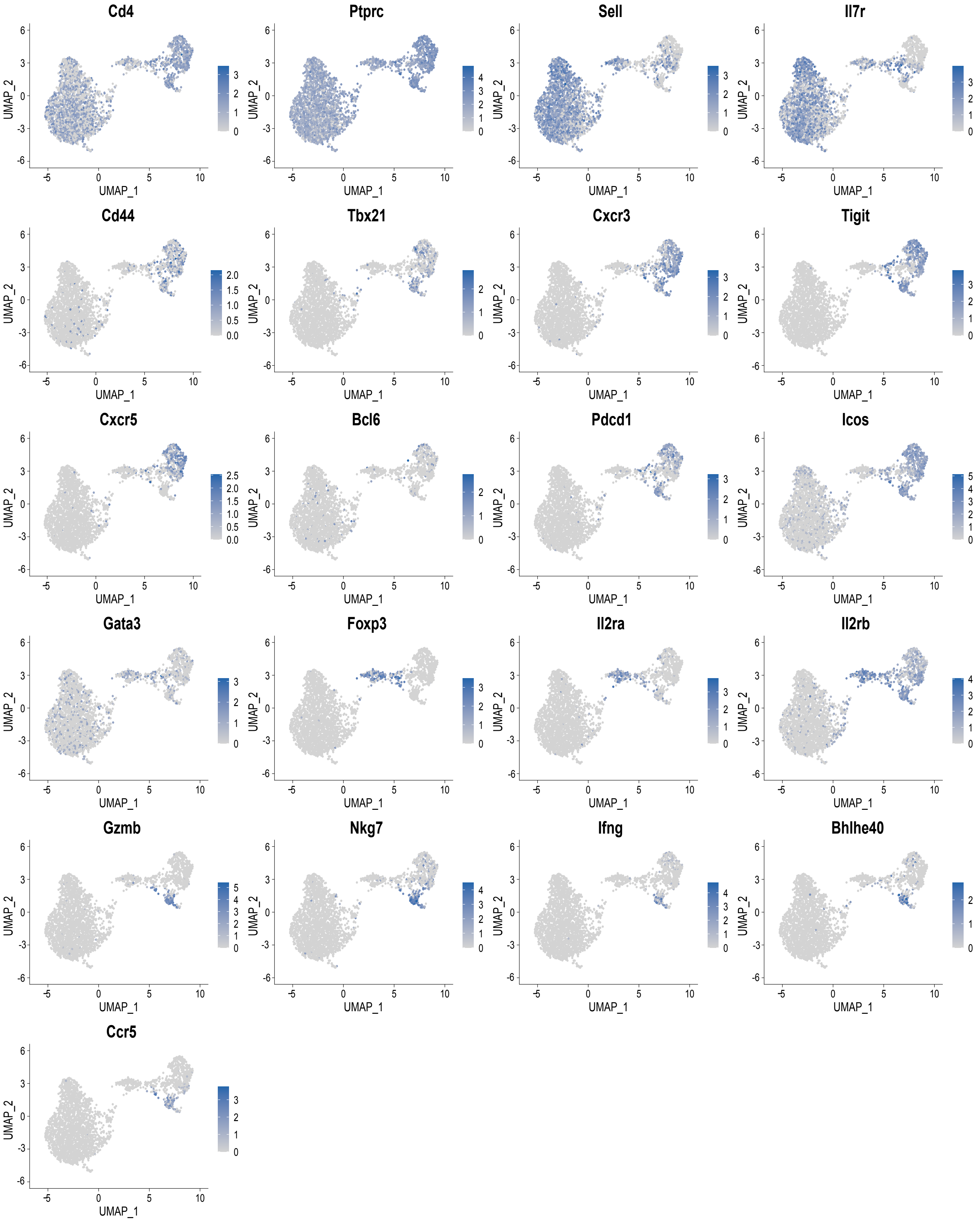

Supplementary Figure 10: Expression of T-cell marker genes of interest, from CD4^{+} splenocytes of 2 challenged mice at day 7 post *P. chabaudi* infection; cell positions are as per UMAP in Figure 8A.

Supplementary Figure 11

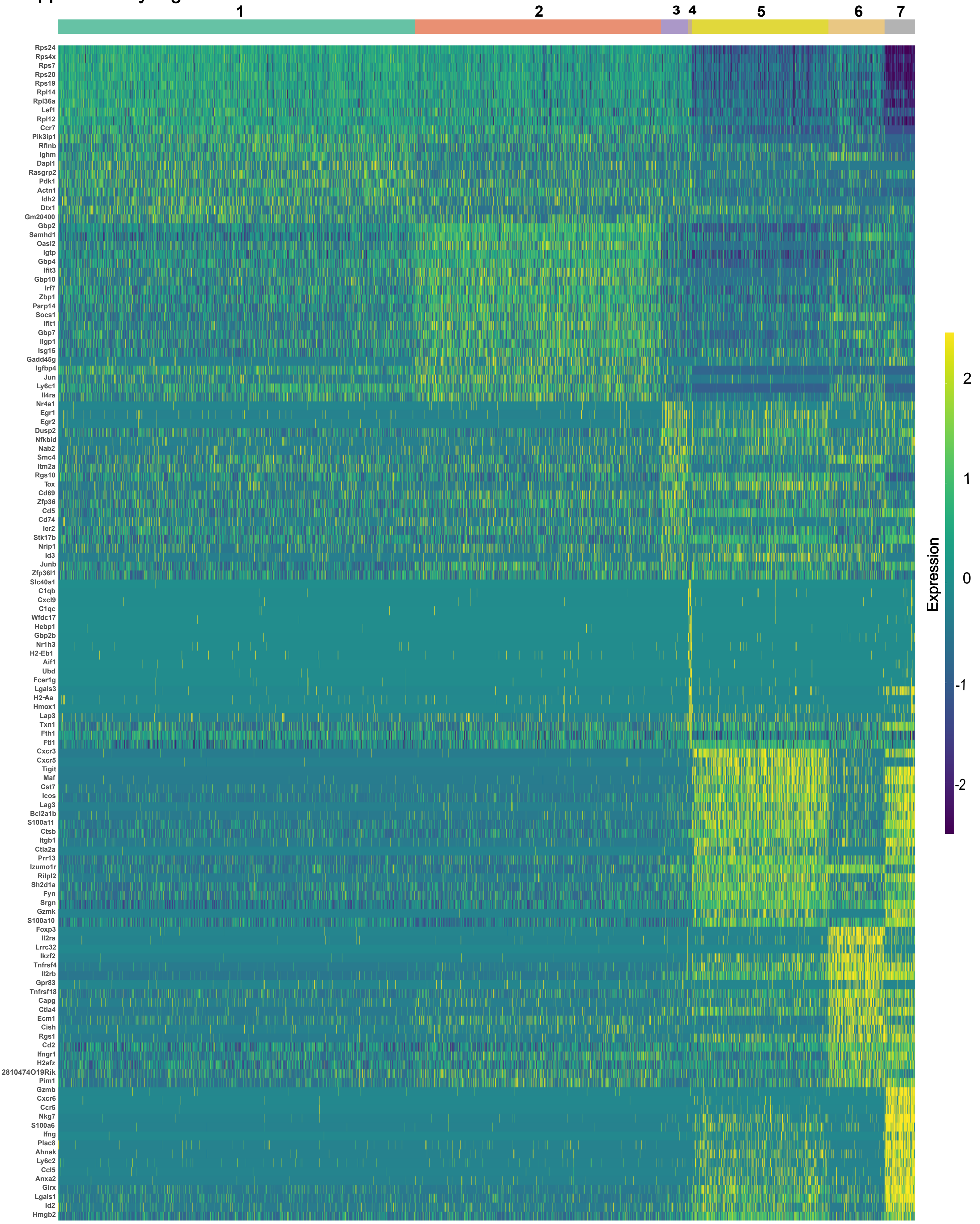

Supplementary Figure 11: D) Heatmap depicts the top 20 differentially expressed genes and their expression values between all 7 distinct clusters, of CD4⁺ splenocytes of 2 challenged mice at day 7 post *P. chabaudi* infection.

Supplementary Figure 12

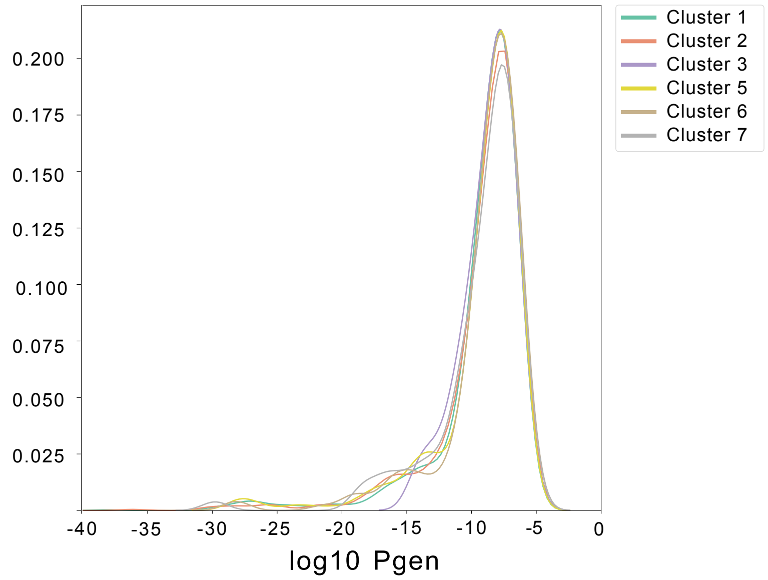

Supplementary Figure 12: KDE plot of the probability of generation (log10) of TCRβ CDR3 nucleotide sequences per cluster, of CD4⁺ splenocytes of 2 challenged mice at day 7 post *P. chabaudi* infection.
