# Supplementary figures and images for: A Conserved TCRβ Signature Dominates a Highly Polyclonal T-Cell Expansion During the Acute Phase of a Murine Malaria Infection
Source: Front Immunol. 2020 Nov 23;11:587756. doi: 10.3389/fimmu.2020.587756 (PMC7719809; doi:10.3389/fimmu.2020.587756)

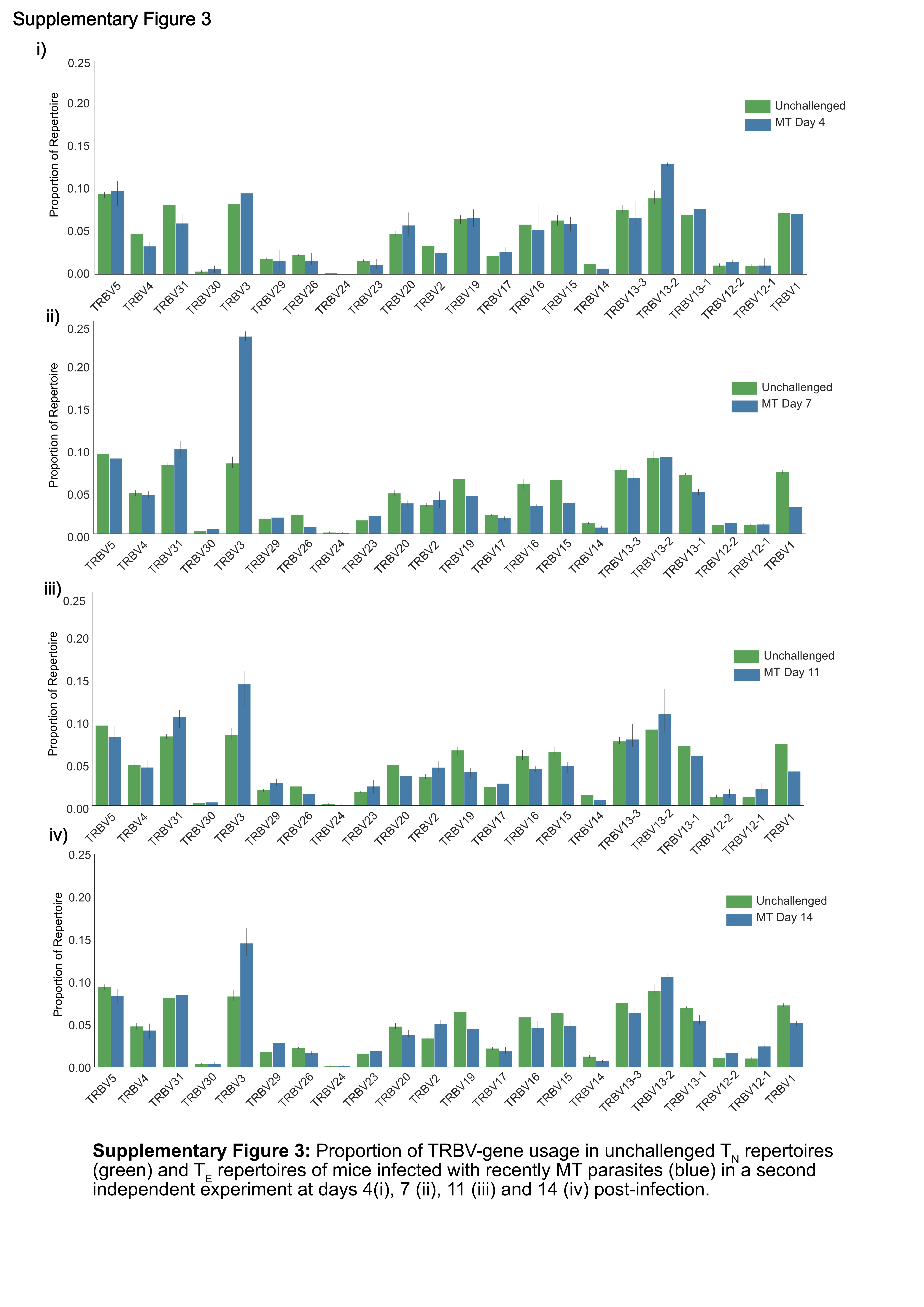

Supplement: Supplementary file 3 [file Image_3.tiff]

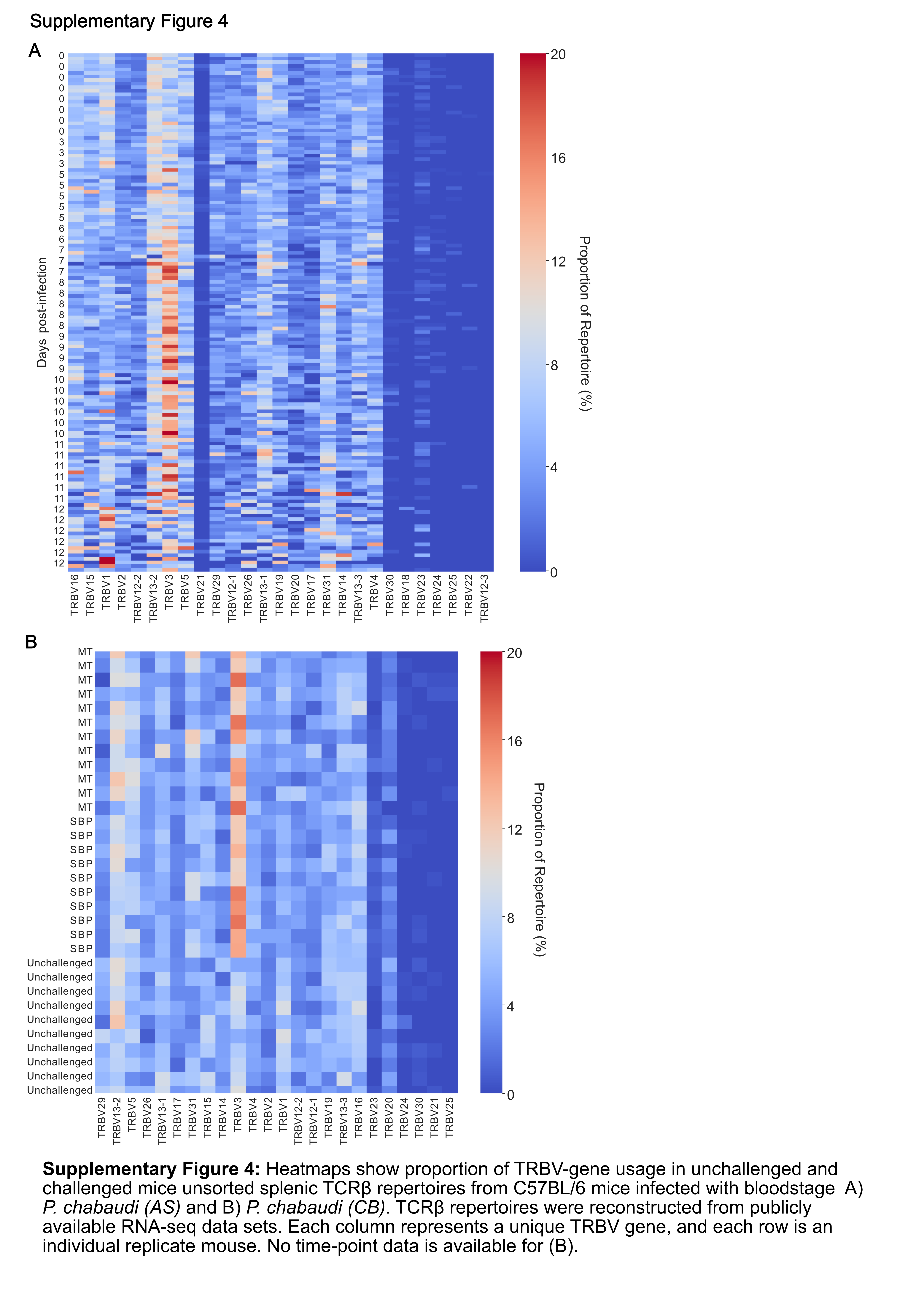

Supplement: Supplementary file 4 [file Image_4.tiff]

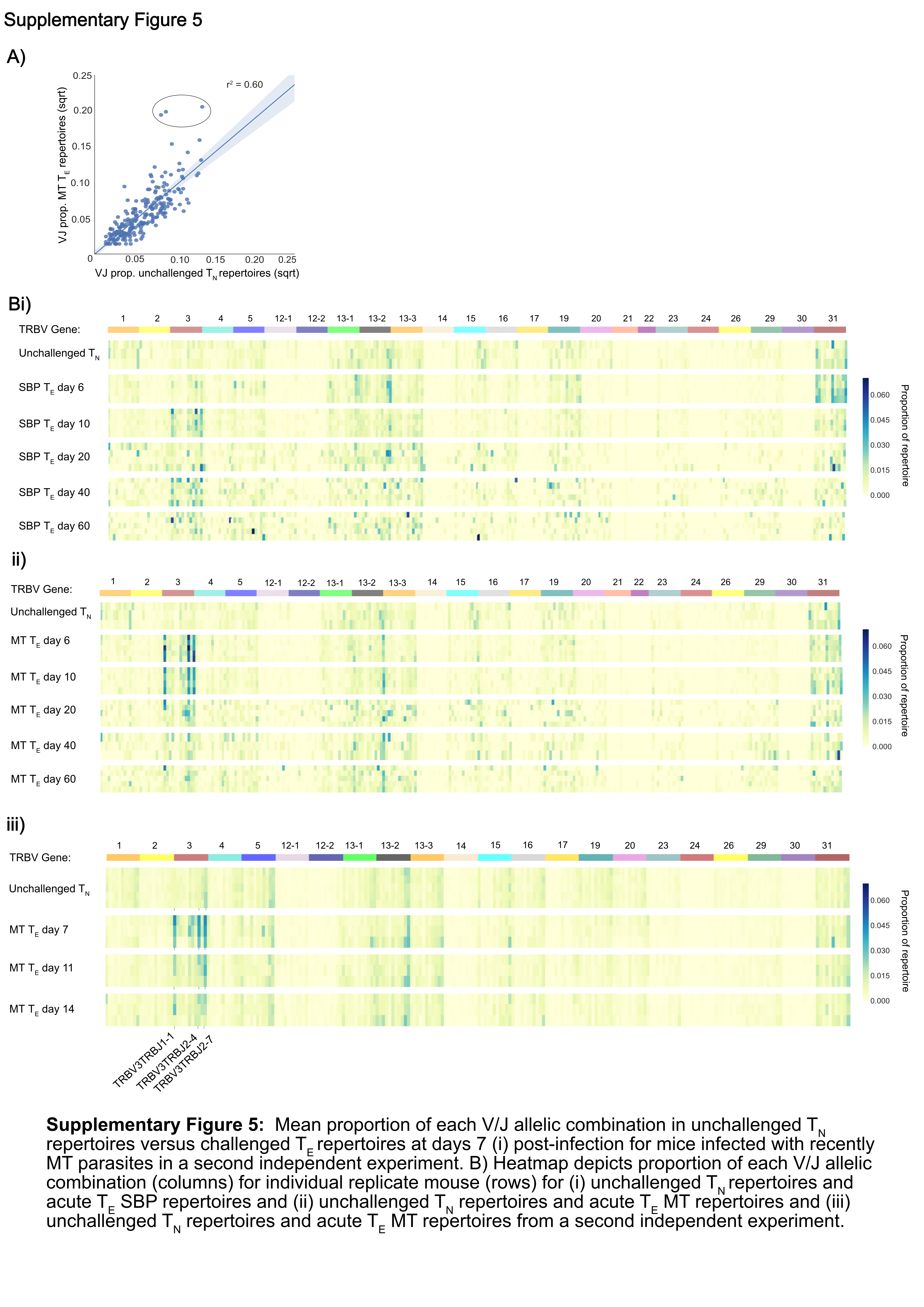

Supplement: Supplementary file 5 [file Image_5.tiff]

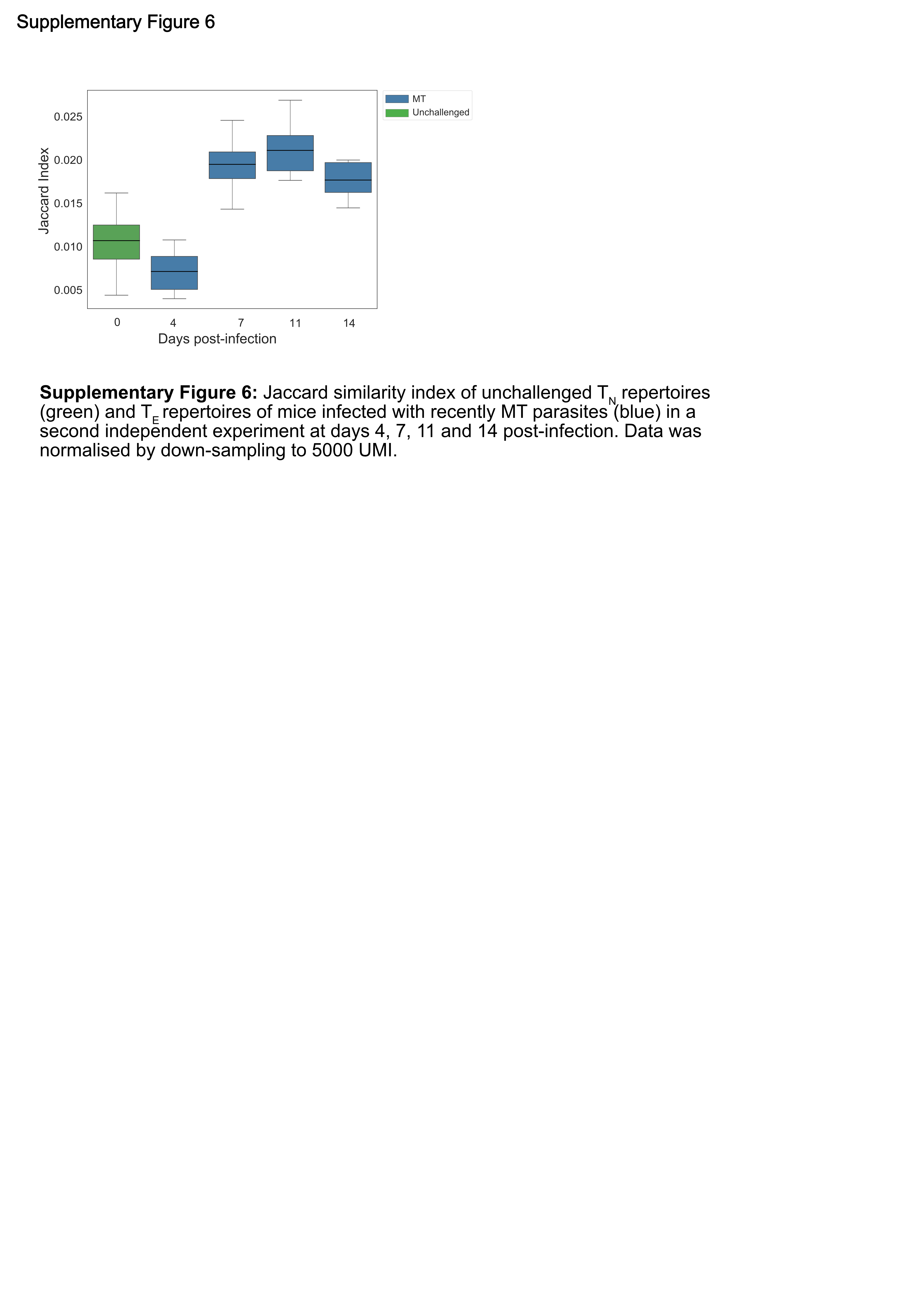

Supplement: Supplementary file 6 [file Image_6.tiff]

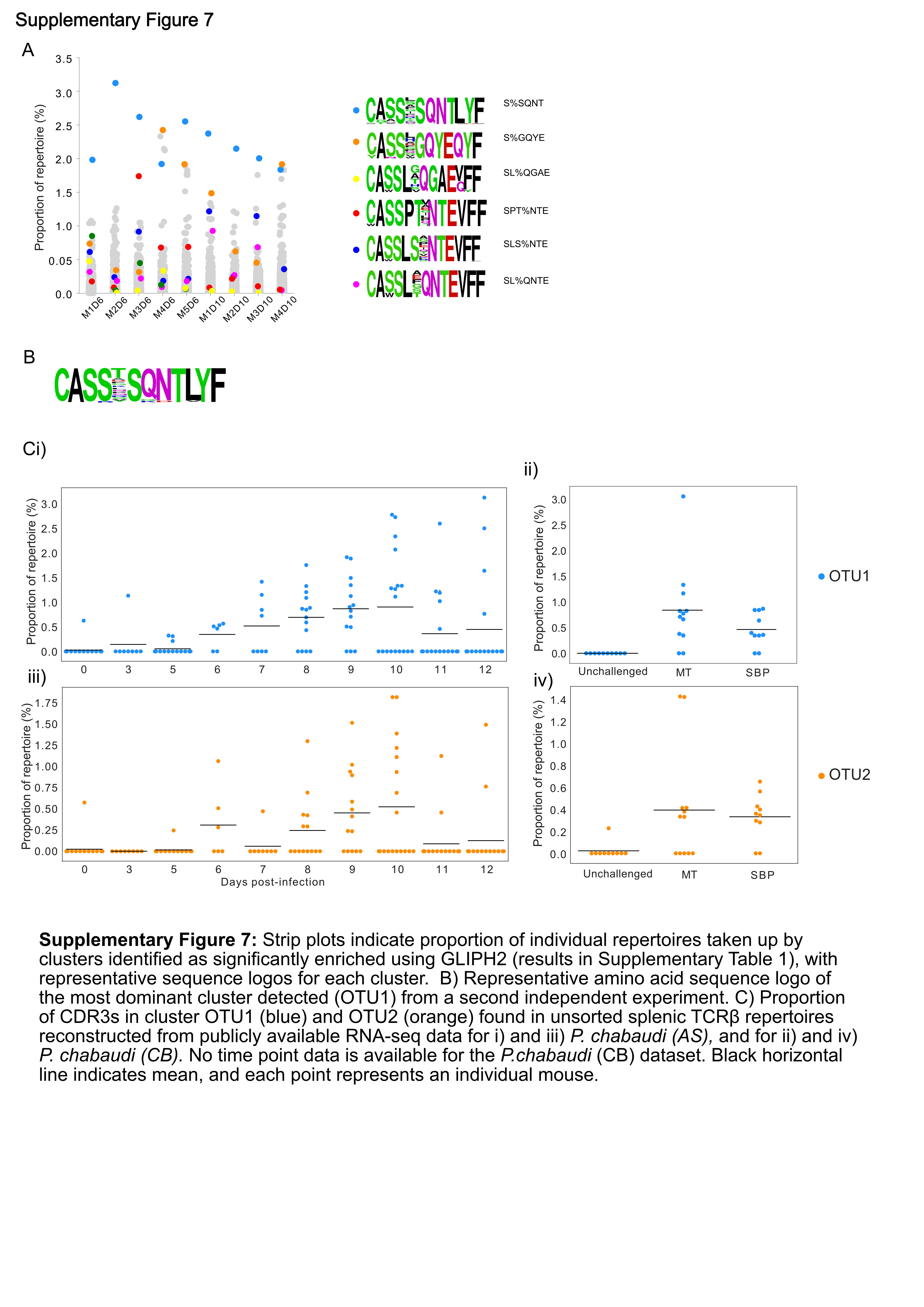

Supplement: Supplementary file 7 [file Image_7.tiff]

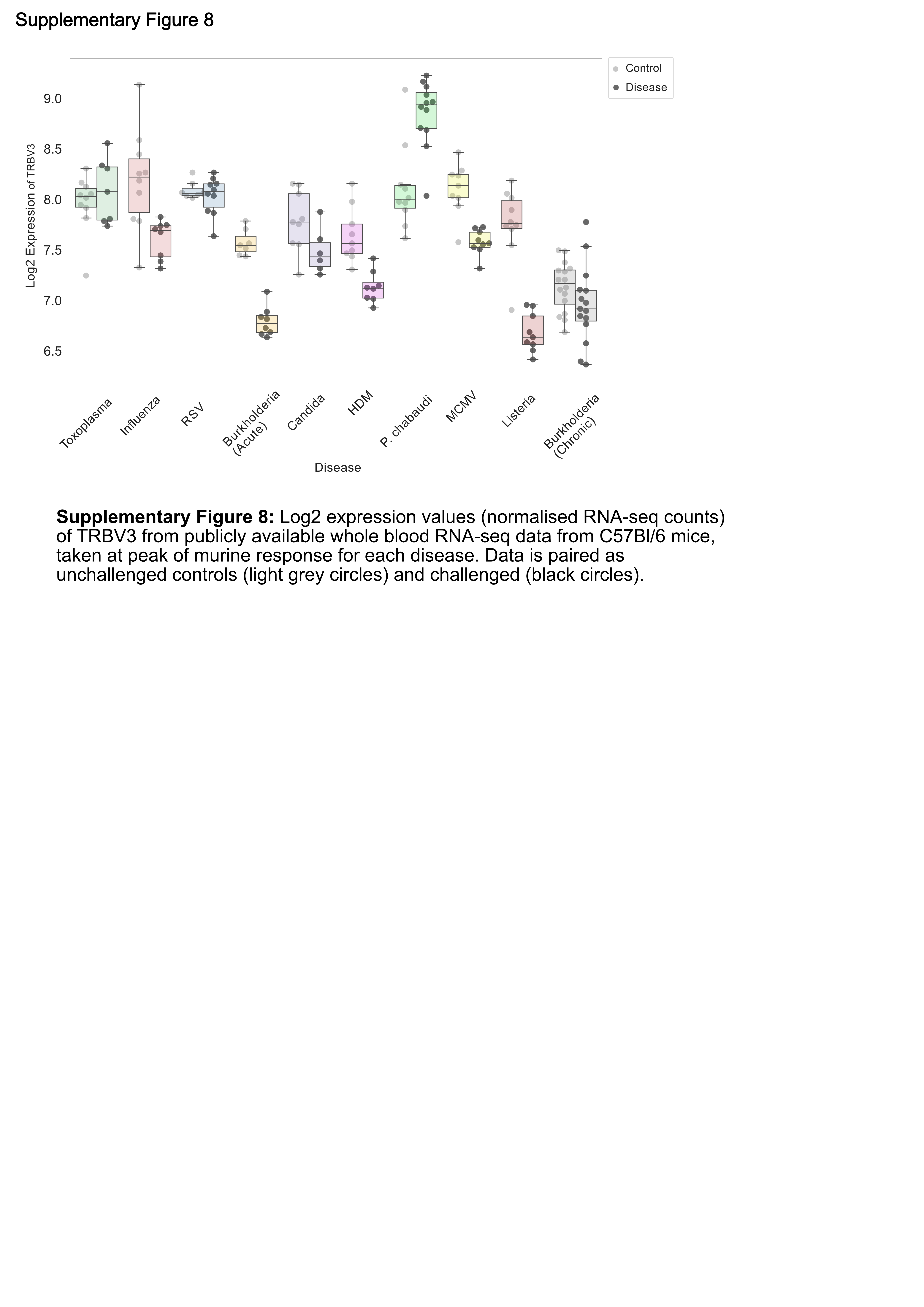

Supplement: Supplementary file 8 [file Image_8.tiff]

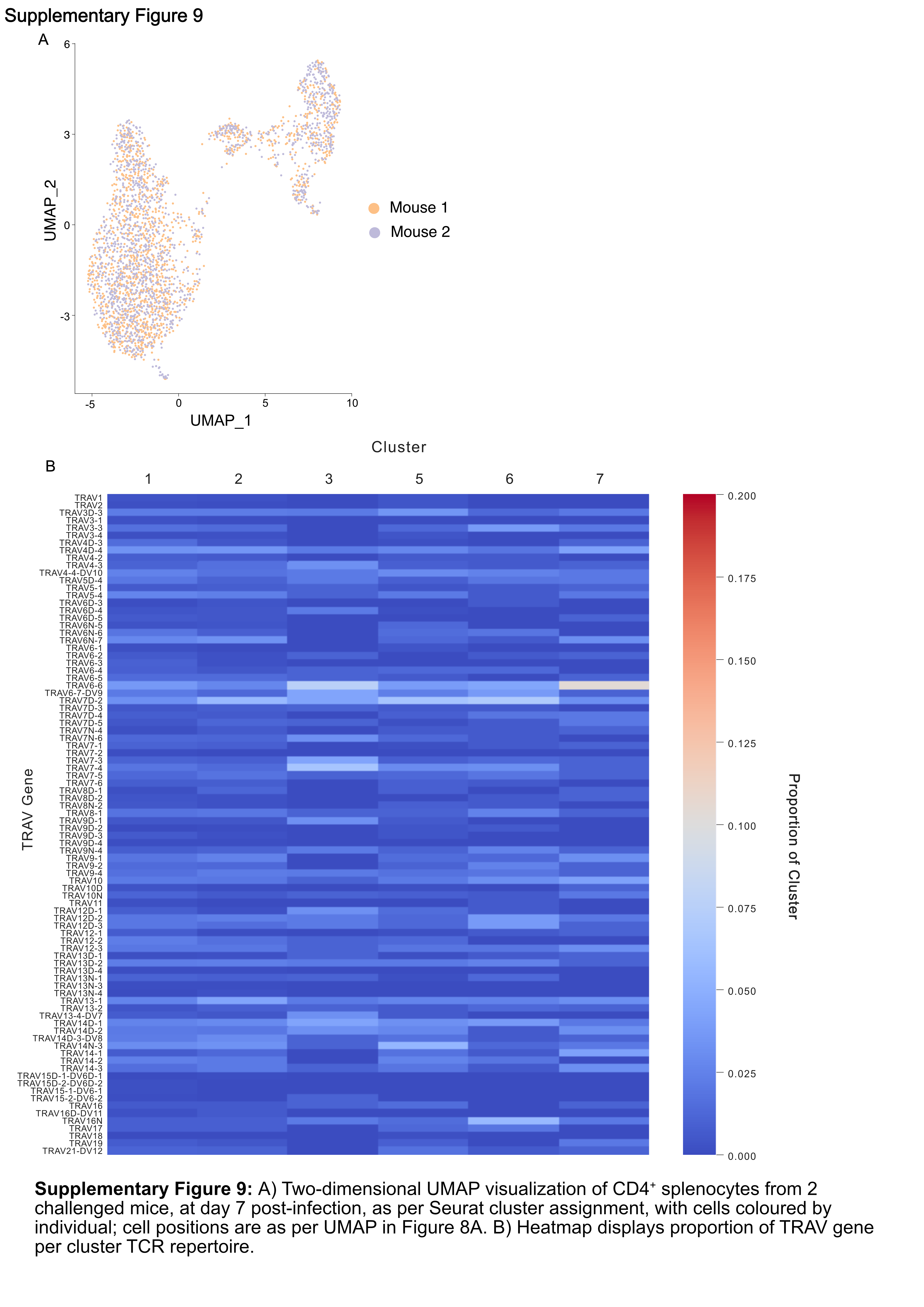

Supplement: Supplementary file 9 [file Image_9.tiff]

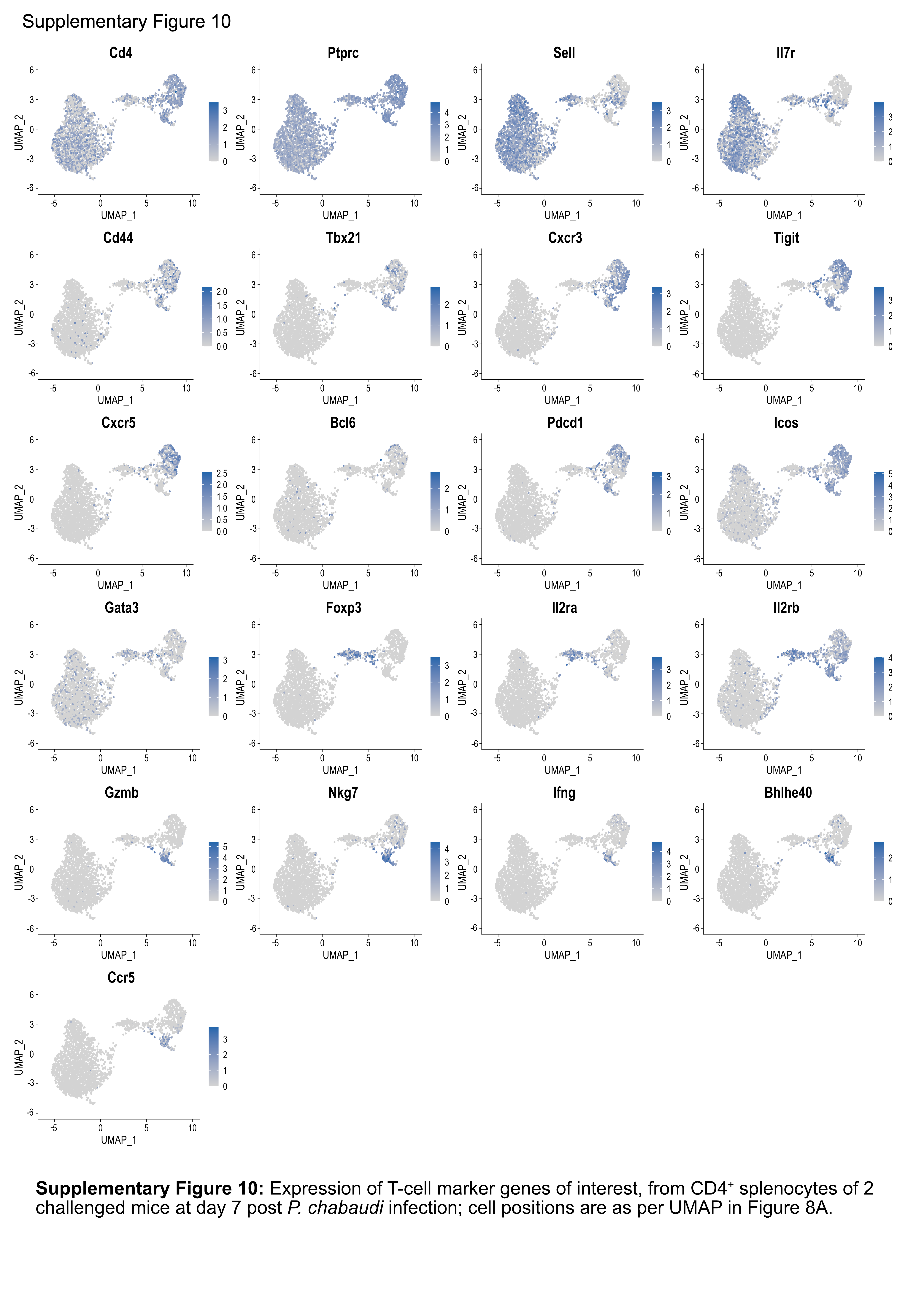

Supplement: Supplementary file 10 [file Image_10.tiff]

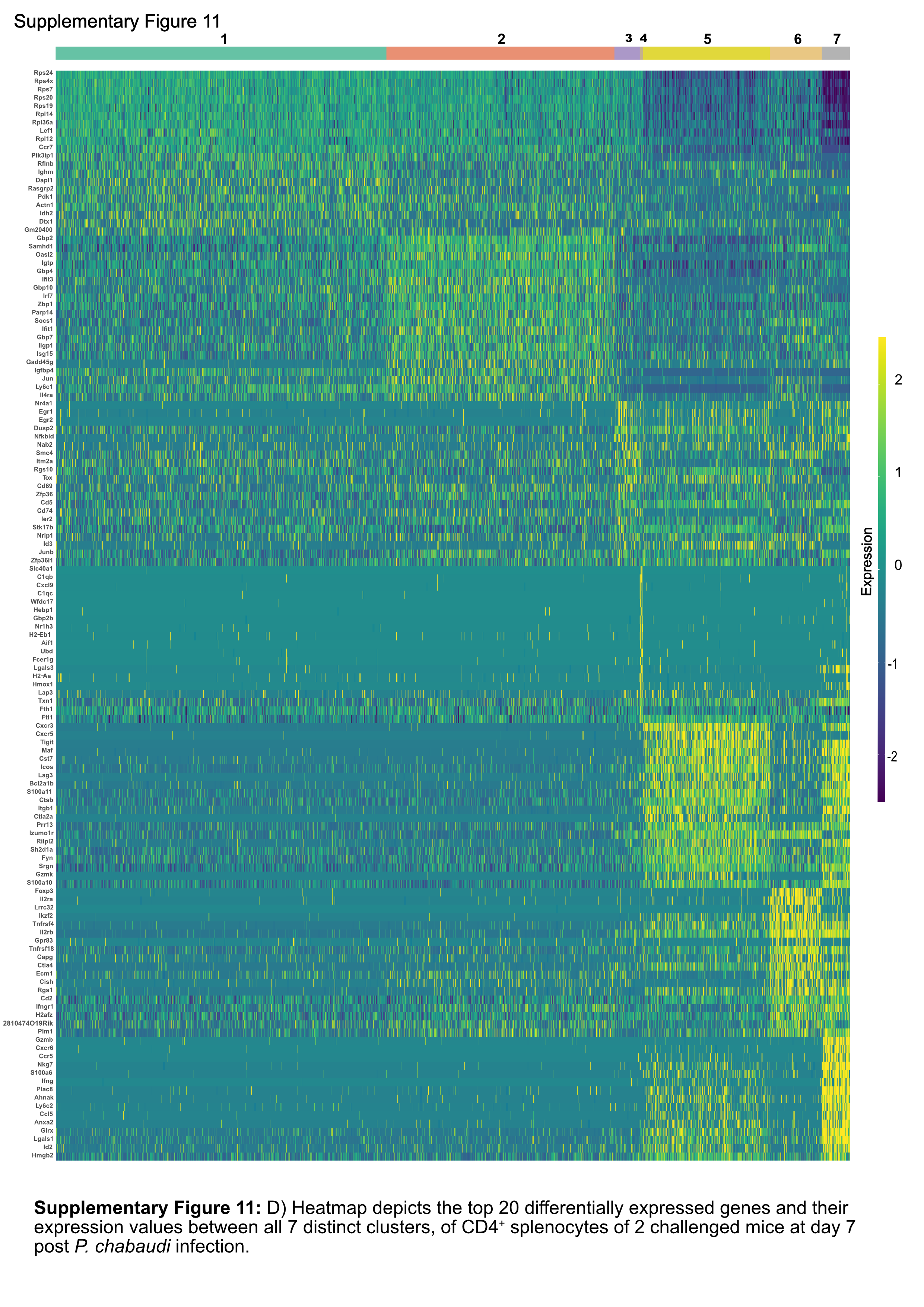

Supplement: Supplementary file 11 [file Image_11.tiff]

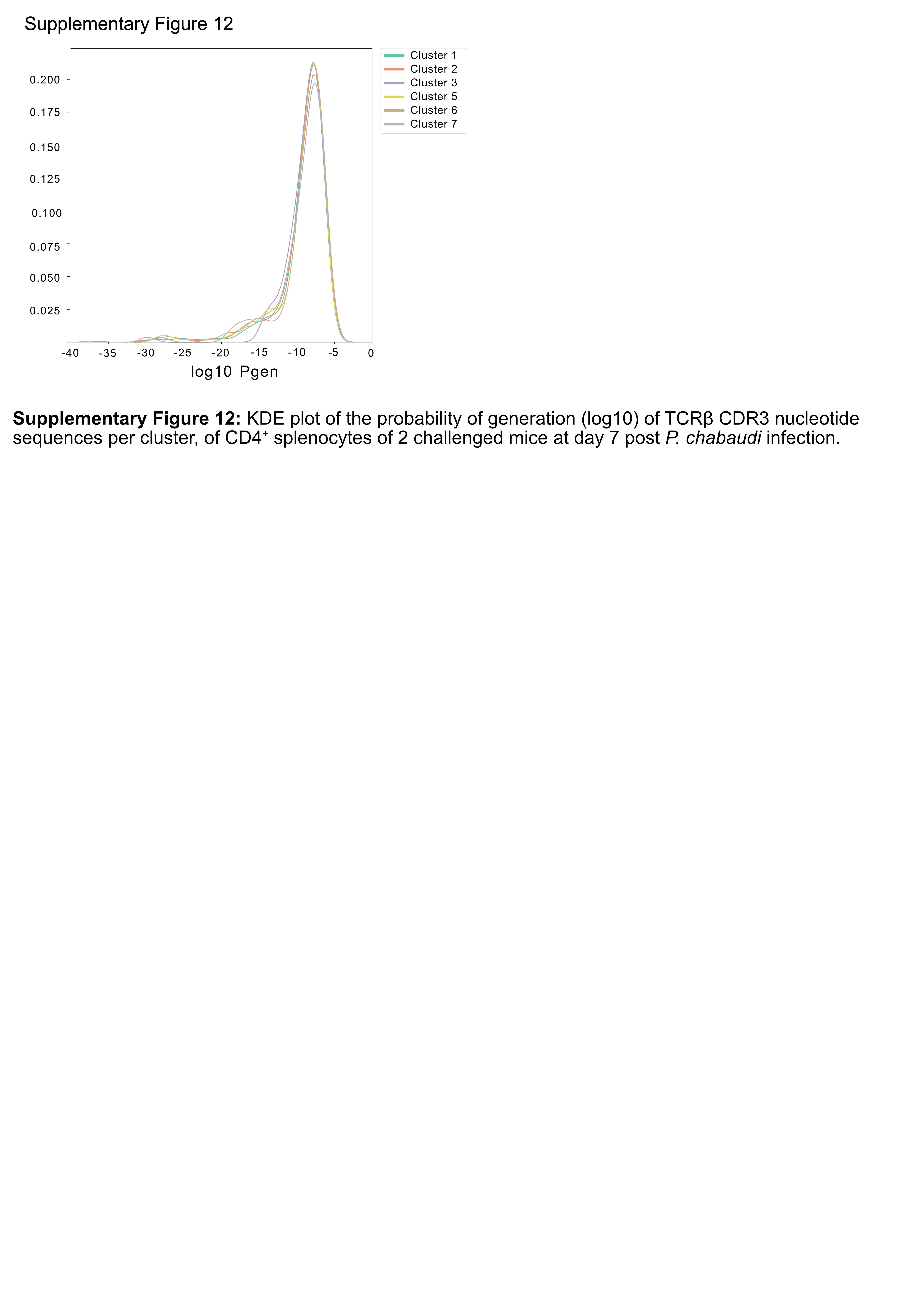

Supplement: Supplementary file 12 [file Image_12.tiff]

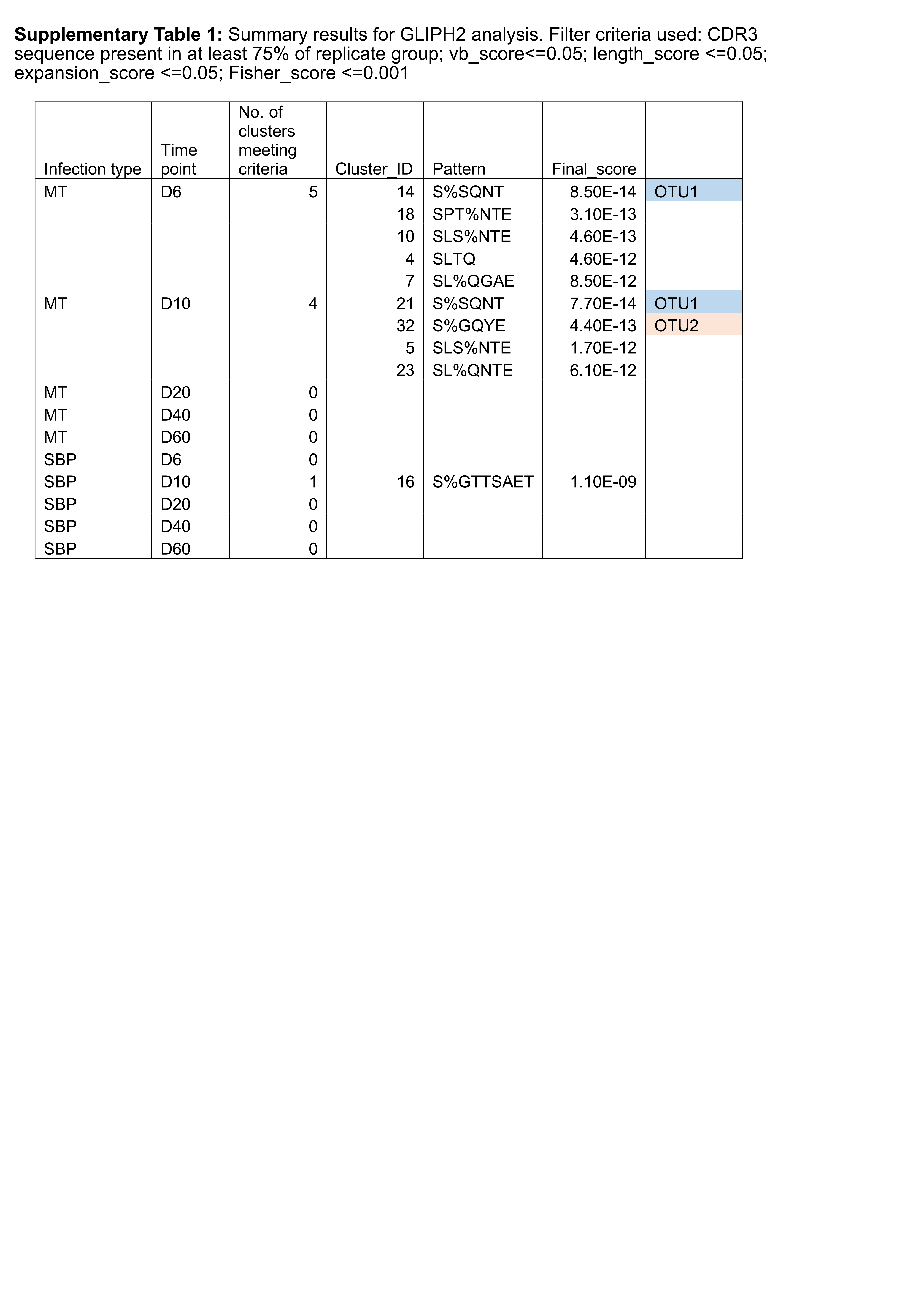

Supplement: Supplementary file 13 [file Image_13.tiff]
